# Sphinganine as a potentially relevant metabolite in pulmonary involvement of primary Sjögren’s syndrome

**DOI:** 10.1016/j.jlr.2025.100961

**Published:** 2025-12-11

**Authors:** Ting Cui, Ziying Geng, Nan Wang, Jing Luo, Zhenyu Li

**Affiliations:** 1 Modern Research Center for Traditional Chinese Medicine of Shanxi University, Taiyuan, China; 2 The Key Laboratory of Chemical Biology and Molecular Engineering of Ministry of Education, Shanxi University, Taiyuan, China; 3 School of Pharmacy, Shanxi Medical University, Taiyuan, China; 4 Department of Rheumatology, The Second Hospital of Shanxi Medical University, Taiyuan, Shanxi, China; 5 Shanxi Key Laboratory of Rheumatism Immune Microecology, Taiyuan, Shanxi, China

**Keywords:** primary Sjögren’s syndrome, pulmonary involvement, sphinganine, ATF6–Myh9 axis

## Abstract

Primary Sjögren’s syndrome (pSS) is a systemic autoimmune disease characterized by lymphocytic infiltration of exocrine glands and frequent extraglandular manifestations, with pulmonary involvement being the most prevalent. However, the mechanisms underlying pulmonary involvement remain unclear, and the role of shared metabolic disturbances in disease pathogenesis is yet to be fully elucidated. Bibliometric analyses have highlighted interstitial lung disease as a key research focus in pSS. In this study, we used an experimental SS mouse model to perform pseudotargeted sphingolipidomics on the salivary glands and lungs. Sphinganine (Sa) was identified as a key metabolite through machine learning-based screening. In vivo experiments demonstrated that administration of Sa aggravated salivary gland injury and pulmonary fibrosis in the experimental SS group. Further in vitro studies revealed that Sa activates the endoplasmic reticulum stress pathway, leading to A253 cell damage and upregulation of fibrosis markers in NIH3T3 fibroblasts. Chemoproteomic analysis revealed that Sa directly binds to the nonmuscle myosin heavy chain IIA (Myh9) and promotes its expression. Pharmacological inhibition of Myh9 restored aquaporin-5 (AQP5) expression in A253 cells and reduced fibronectin and alpha-smooth muscle actin levels in NIH3T3 cells. Collectively, this study indicates that Sa, as a shared regulatory metabolite between the salivary gland and lung, appears to be implicated in the ATF6–Myh9 signaling axis and may contribute to pSS-related pulmonary injury. Nevertheless, this relationship warrants further validation in future studies. In parallel, it proposes a novel strategy for identifying common metabolic biomarkers across affected organs in autoimmune diseases.

Primary Sjögren's syndrome (pSS) is an autoimmune disorder affecting connective tissue, primarily impairing exocrine glands such as salivary and lacrimal glands, resulting in xerostomia and keratoconjunctivitis sicca (1). In addition to exocrine gland involvement, pSS frequently affects multiple extraglandular organs, with pulmonary involvement being particularly common. This includes airway lesions, lymphoproliferative disorders, and interstitial lung disease (2). Although the pathogenic mechanisms of pSS have been extensively studied in terms of immunology and genetics, research remains limited due to the disease’s complexity.

Metabolomics represents a burgeoning field within omics technologies, characterized by its high sensitivity and resolution, extensive dynamic range, and cost-effectiveness, all aimed at investigating small molecules and metabolic pathways in organisms. Metabolomic analyses of biological fluids such as saliva (3), tear (4), serum (5), and urine (6) have identified metabolic disturbances associated with pSS and have contributed to the development of metabolite-based diagnostic models (7). Despite these advancements, the biological roles of metabolites in pSS are not yet fully elucidated. The progression of functional metabolomics has shifted research on metabolite biomarkers from mere descriptive profiling to a focus on mechanistic understanding and translational applications. In the context of pSS, the short-chain fatty acid butyrate has been demonstrated to have immunoregulatory effects through the modulation of B cell function (8). Notably, extraglandular tissues such as the lungs often display histopathological features similar to those observed in salivary glands, including lymphocytic and mononuclear cell infiltration around ducts or intact glands, as well as in adjacent epithelial structures (9). This cross-organ pathological resemblance suggests the presence of a shared regulatory mechanism. However, the specific molecular basis underlying this phenomenon remains unclear, highlighting the need for further functional studies to uncover how metabolites contribute to multiorgan involvement in pSS.

To address the research gap concerning metabolic alterations associated with organ involvement in pSS, we developed a comprehensive and systematic strategy for metabolite biomarker discovery. The workflow begins with bibliometric analysis to define research priorities, followed by tissue-specific lipidomic profiling of both salivary gland and lung organs. Key metabolites are then prioritized using machine learning algorithms and subsequently validated through in vivo and in vitro experiments. Finally, chemoproteomic approaches are employed to identify potential protein targets of key metabolites and to confirm their binding interactions and functional relevance. This integrated strategy provides a robust framework for elucidating how metabolites mediate organ involvement in pSS (Fig. 1).

**Fig. 1:**
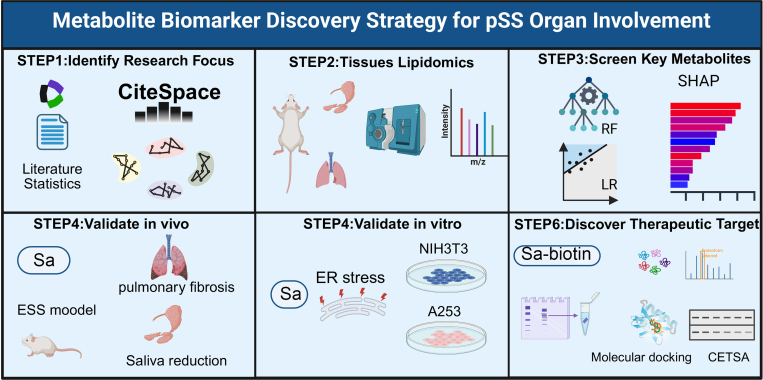
Workflow of the study. Step 1: Identification of research focus through bibliometric analysis; Tissue-specific; Step 2: lipidomic profiling of salivary glands and lungs; Step 3: Screening and prioritization of key metabolites using machine learning models; Step 4: In vivo validation using the ESS mouse model; Step 5: In vitro functional validation in A253 and NIH3T3 cells; Step 6: Chemoproteomic analysis and validation of binding and functional roles of key protein targets. ESS, experimental Sjögren’s syndrome.

## Materials and methods

### Bibliometric and visualization analysis

Using the keywords “Sjögren's syndrome” and “lung,” a systematic search was carried out in the Web of Science (WOS) Core Collection to collect publications from 2015 to 2025. The inclusion criteria required articles to be peer-reviewed and either original research or reviews. Exclusion criteria included conference abstracts, errata, unpublished articles, duplicate publications, and irrelevant studies. CiteSpace (version 6.4 R6), a widely used Java-based bibliometric tool, was employed to analyze the knowledge structure, distribution patterns, and temporal trends in this research field (10). The analyses included the distribution of publications by country/region, keyword clustering, and the temporal evolution of research themes.

### Establishment and treatment of ESS in mice

Eight-week-old BALB/c female mice, weighing 18–20 g, were obtained from Beijing Vital River Laboratory Animal Technology Co., Ltd. (License No. SCXK 2016-0006, Beijing, China). The Animal Ethics Committee of Shanxi University approved all experimental procedures. Following the earlier description, experimental SS (ESS) induction was executed. Briefly, salivary glands were collected, processed in PBS, and centrifuged to retrieve salivary gland proteins. Proteins, quantified via the BCA assay, were diluted to 5 mg/ml and mixed with an equal volume of complete Freund’s adjuvant, achieving a final concentration of 2.5 mg/ml. Mice were subcutaneously injected with the emulsions on their backs on days 0 and 7. On day 14, a booster immunization was administered with Freund’s incomplete adjuvant combined with salivary gland proteins at the same concentration. Successful model establishment was defined by salivary flow rates <30 mg/10 min. Mice were randomly divided into three groups: Control, ESS, and ESS + sphinganine (Sa) (20 mg/kg). Sa was dissolved in a vehicle composed of 10% DMSO and 90% saline (v/v, 1:9) and administered intraperitoneally at a dose of 20 mg/kg once daily for two consecutive weeks.

### Salivary flow rate and mouse weight and organs indexes

Initially, mice were induced to secrete saliva for 5 min by intraperitoneal injection of 0.1 mg/kg pilocarpine, followed by anesthesia with isoflurane (3% for induction and 2% for maintenance) in an anesthesia machine. Preweighed cotton balls were placed under the tongues of the mice for 10 min. Saliva weight was determined by measuring the weight difference of cotton balls before and after soaking. At the conclusion of the experiment, salivary glands and lungs were collected, and organ indexes were determined by calculating (organ weight/body weight) × 100%.

### Histological evaluation of lung tissues

The left lung tissues were fixed in paraformaldehyde, dehydrated with graded ethanol, and embedded in paraffin. Paraffin-embedded tissues were sectioned at 4 μm thickness. Servicebio Co. Ltd. (Wuhan, China) conducted the hematoxylin and eosin (H&E) staining. Sirius red staining was conducted using the Masson Stain Kit (G1472, Solarbio, China), while Masson’s trichrome staining was carried out with the Masson Stain Kit (G1346, Solarbio, China). During immunohistochemistry, sections were treated with an anti-fibronectin (FN) antibody at 4°C overnight, washed thrice, and incubated with an HRP-conjugated secondary antibody. The stained sections were viewed and digitally captured through a light microscope.

### Pseudotargeted sphingolipids analysis by LC-MS/MS

Samples of salivary glands and lungs were prepared following the method outlined in the literature (11). Twenty milligram sample of submandibular gland tissue was taken and added to 0.4 ml of precooled chloroform/methanol extraction reagent (2:1, v/v). The sample was vortexed for 10 min and then spun at 12,000 rpm for 10 min at 4°C. The bottom phase was transferred to a fresh 1.5 ml centrifuge tube and evaporated using a nitrogen stream. For metabolomic analysis, the residue was redissolved in 100 μl of cold methanol/isopropanol (1:4, v/v) and centrifuged at 12,000 rpm for 10 min to obtain the supernatant. The mobile phase system comprises methanol/water (30:70) with 0.2% formic acid (A) and methanol/isopropanol (70:30) with 0.02% formic acid (B), operating at a flow rate of 0.4 ml/min. Mass spectrometric analysis was conducted in positive ion mode using multiple reaction monitoring (MRM). The ion source parameters were set as follows: ion spray voltage +5500 V, source temperature 550 °C, Gas Source 1 = 55 psi, Gas Source 2 = 55 psi, curtain gas = 35 psi. MRM ion pairs, declustering potential (DP), and collision energies (CE) for each targeted metabolite are summarized in Supplementary Table S3. Precursor and product ions for sphingolipid identification were selected with reference to published studies and further optimized on our instrument.

### Screening metabolite features based on SHAP values

This study utilized logistic regression (LR) and random forest (RF) algorithms to create diagnostic models from metabolomics profiles. The LR model estimates the probability of disease as a linear combination of metabolite features, whereas the RF model utilizes an ensemble of decision trees to capture complex nonlinear associations and improve model robustness.

Following model training, Shapley additive explanations (SHAP) were utilized to elucidate the impact of individual metabolites (12). The SHAP analysis includes five steps: generating feature combinations, calculating marginal contributions, computing weighted averages, interpreting individual predictions, and assessing global feature importance. SHAP values were derived using the TreeExplainer algorithm from the SHAP Python package alongside scikit-learn.

For any two features m and n, the interaction SHAP value $\phi_{m,n}$ is calculated as follows:

$$\phi_{m,n}=\sum_{R\subseteq P\left\{m,n\right\}}\frac{\left|R\right|!\left(N-\left|R\right|-2!\right)}{2\left(N-1\right)!}\varepsilon_{mn}\left(R\right)$$

where N is the full set of features, R represents any subset not containing m and n, and $\varepsilon_{mn}\left(R\right)$ denotes the marginal contribution of the interaction between m and n under subset R, defined as

$$m\neq n,\varepsilon_{mn}\left(R\right)=gx\left(R\cup \left\{m,n\right\}\right)-gx\left(R\cup \left\{m\right\}\right)-\left(R\cup \left\{n\right\}\right)+gx\left(R\right)$$

Here, gx(∙) represents the model output for a given feature subset. This calculation systematically incorporates feature interaction effects, allowing the identification of synergistic or redundant contributions among metabolites in the prediction model, thereby improving the accuracy and robustness of feature importance interpretation.

### Sample collection and clinical stratification

Eighty-three serum samples were collected, comprising 49 patients diagnosed with pSS based on the 2016 ACR/EULAR criteria and 34 age- and sex-matched healthy controls (HCs). The study received approval from the institutional ethics committee (2022YX063), and all participants provided consent. This study was conducted following the principles of the Declaration of Helsinki principles. Chi-square and independent *t*-tests were used to analyze age and sex demographics between the two groups to ensure baseline comparability. Detailed information is provided in Supplementary Table S2.

### Targeted LC-MS/MS analysis of Sa

Sample pretreatment was performed according to the pseudotargeted lipidomics protocol. Sa quantification was performed using a Shimadzu LC-20A system paired with an AB Sciex QTRAP 4500 triple quadrupole mass spectrometer. Chromatographic separation was conducted using a 37°C ACQUITY UPLC BEH C18 column (1.7 μm, 2.1 × 100 mm, Waters, Ireland). The mobile phase used solvent A, which was 0.2% formic acid in methanol and water (3:7, v/v), and solvent B, which was 0.02% formic acid in methanol and isopropanol (7:3, v/v), at a flow rate of 0.3 ml/min. The linear gradient elution was programmed as follows: solvent B was increased from 85% to 100% over 0–3 min, held at 100% from 3–6 min, and then returned to 85% over 0.5 min for column re-equilibration. Data acquisition utilized positive ion mode with MS parameters optimized for pseudotargeted lipidomics. Quantification of Sa(d18:0) was performed using an external standard calibration curve. A series of standard solutions (e.g., 0.5–500 ng/ml) were prepared and analyzed under the same LC-MS/MS conditions as the biological samples. The calibration curve was constructed by plotting peak area versus concentration, and linear regression yielded the equation y = 0.0015x – 0.0003 with a correlation coefficient (*R*^2^ = 0.9989) (Supplementary Fig. S2). The concentration of Sa(d18:0) in tissue and serum samples was calculated based on the calibration curve. Data analysis was conducted using MultiQuant 2.1 software (Sciex, Foster City, CA).

### Cell culture and inhibitor treatment

The A253 human submandibular gland tumor cell line (Servicebio, Wuhan, China) was cultured in McCoy's 5A medium (Gibco, New York). The NIH3T3 mouse embryonic fibroblast cell line (Pricella, Wuhan, China) was cultured in DMEM (Gibco, New York). Cell lines were maintained at 37°C in a humidified 5% CO_2_ atmosphere, using a culture medium supplemented with 10% fetal bovine serum, 100 U/ml penicillin, and 100 μg/ml streptomycin. All experiments utilized cells in the exponential growth phase. In the endoplasmic reticulum (ER) stress inhibition assay, NIH3T3 cells underwent pretreatment with 5 μM 4-phenylbutyric acid (4-PBA, MedChem Express) for 4 h in a serum-free medium. For the Myh9 inhibition assay, cells were pretreated with 2 μM Blebbistatin (TargetMol, Shanghai, China) for 4 h in serum-free medium.

### Sa-biotin pull-down assays

Upon reaching 80% confluency, cells were washed with PBS and incubated for 4 h at 37°C with 5% CO_2_ using either 10 μg of Sa-biotin probe (Echelon Biosciences, #S-110B) or control biotin (Sigma-Aldrich, #B4501). The Sa-biotin probe was initially dissolved in DMSO and then diluted in serum-free medium immediately before use to ensure solubility. Following incubation, cells were lysed in RIPA buffer supplemented with 1% EDTA-free protease inhibitor cocktail. Supernatants were collected after centrifugation at 12,000 g for 15 min at 4°C. Biotin-labeled proteins were enriched by incubation with Dynabeads™ M-280 streptavidin magnetic beads (Thermo Fisher Scientific, #11206) under gentle rotation for 9 h at 4°C. To reduce nonspecific binding, beads were washed five times with TBST (TBS containing 0.1% Tween-20) followed by two washes with high-salt buffer (500 mM NaCl). Protein-bound fractions were eluted and separated by SDS-PAGE, followed by western blotting or Coomassie brilliant blue staining. Protein bands (∼40 kDa) were excised, stored in deionized water, and submitted to Shanghai Applied Protein Technology Co., Ltd. for identification and quantification.

### Cellular thermal shift assay

A253 or NIH3T3 cells were planted in 10-cm dishes and subjected to Sa or a vehicle control for 4 h. The cells were then gathered, rinsed with PBS, and resuspended in PBS containing protease inhibitors. Cell suspensions were aliquoted into PCR tubes, subjected to 3-min heat treatments at temperatures between 43°C and 61°C and subsequently cooled to room temperature. After freeze-thaw lysis, samples were centrifuged at 20,000 g for 20 min at 4°C. Western blot analysis was performed on the supernatants using an anti-Myh9 antibody. Band intensities were normalized to the unheated control for quantification.

### Statistical analysis

Data analysis was conducted using GraphPad Prism 8 (GraphPad, La Jolla, CA), with results presented as mean ± SD. Pairwise comparisons were conducted using a two-tailed Student's *t*-test, while one-way ANOVA was utilized for comparisons among multiple groups. A *P* value of less than 0.05 was considered statistically significant.

## Results

### Lung involvement is a major focus in pSS research

**Fig. 2:**
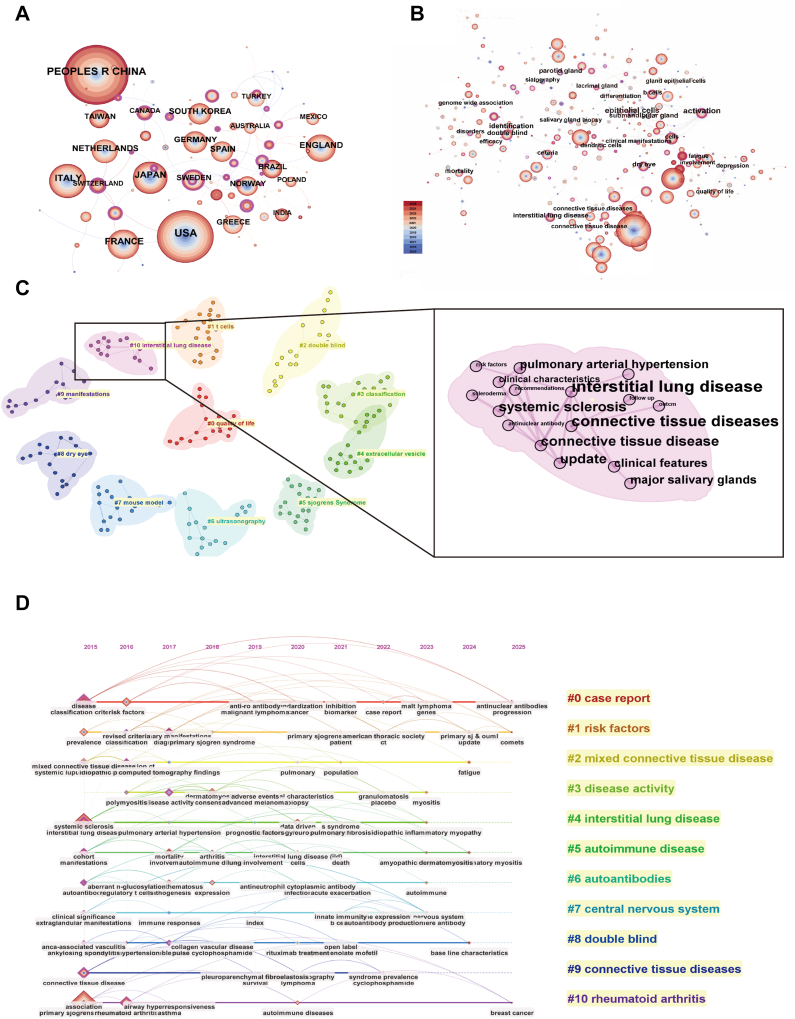
Analysis and visualization of research hotspots of pSS. A: Cooperation network map of countries. The size of each node represents the number of publications, and the connecting lines indicate international collaboration. Different node colors correspond to the year of cooperation; B: Co-occurrence network map of high-frequency keywords; C: Keyword cluster analysis. Cluster labels were extracted using the Latent Semantic Indexing (LSI) algorithm and grouped into 11 distinct clusters; D: Timeline view of keyword clustering related to pSS and lung. Each horizontal line represents a keyword cluster, with colored nodes denoting individual keywords. The position of a node reflects the year in which the corresponding keyword first appeared in the literature. pSS, primary Sjögren’s syndrome.

Initially, a bibliometric analysis was conducted using “primary Sjögren’s syndrome” as the keyword in the WOS database, resulting in the inclusion of 5,370 relevant publications. CiteSpace 6.4.R6 was employed for visualization and comprehensive analysis to elucidate the overall development trends and key research area in pSS. In terms of publication volume by country or region, China ranked first with 1,260 publications, followed by the United States with 970. These two countries represent major contributors to pSS research (Fig. 2A). Keyword cluster analysis revealed 11 distinct research clusters, with a modularity Q value of 0.8471 and an average silhouette score of 0.9487, both satisfying the reliability criteria for clustering analysis. The frequency and prominence of research on interstitial lung disease, a major extraglandular organ involvement, have significantly increased, indicating that lung involvement has emerged as a key focus in pSS research (Fig. 2B, C).

To further investigate the research landscape of pSS with pulmonary involvement, 427 publications containing both “primary Sjögren’s syndrome” and “lung” as keywords were selected for keyword clustering and timeline analysis. The visualization reveals that predominant research themes include “case reports,” “risk factors,” and potential associations with other autoimmune diseases such as central nervous system and rheumatoid arthritis (Fig. 2D). However, the underlying pathogenesis of pSS with pulmonary involvement remains poorly understood, particularly with respect to shared metabolic alterations underlying multiorgan involvement.

### The ESS mouse model was constructed and accompanied by lung fibrosis

**Fig. 3:**
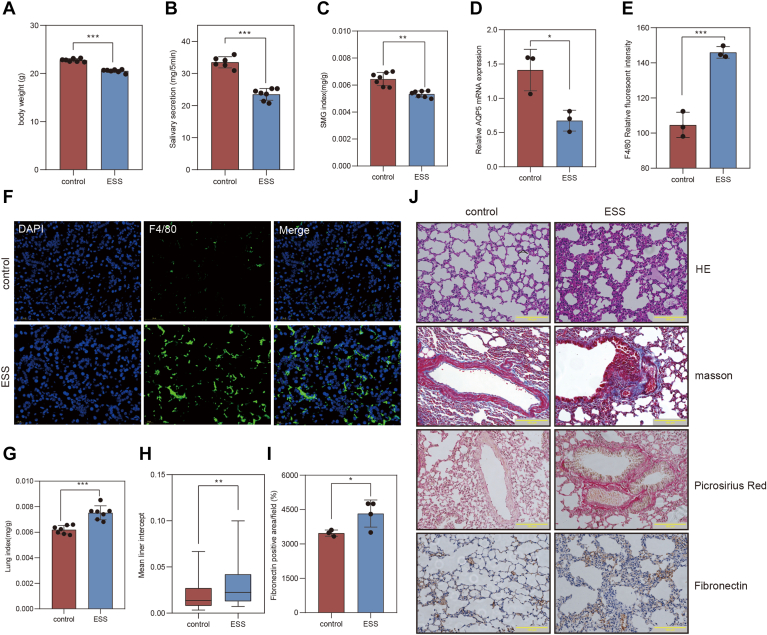
Characterization of the ESS mouse model with pulmonary involvement. A–C: Comparisons of body weight (A), salivary flow rate (B), and salivary gland index (C) between control and ESS groups; (D) Relative mRNA expression level of *Aqp5* in salivary gland tissues; (E) Quantification of F4/80^+^ macrophage infiltration in the salivary gland based on relative fluorescence intensity; (F) Immunofluorescence staining of salivary glands showing F4/80^+^ macrophage infiltration (green); nucleus was counterstained with DAPI (blue). G: Lung index was calculated. Lung index = lung weight (g)/body weight (g) × 100%. H: The mean linear intercept of lung tissues from each group. I: Quantification of fibronectin-positive area (%) in lung. J: Representative histological staining of lung tissues: H&E staining, Sirius red staining for collagen deposition, Masson’s trichrome staining highlighting collagen (blue) and muscle fibers (red), and immunohistochemical staining of fibronectin. Data are presented as mean ± SD. *P* < 0.05, *P* < 0.01, ∗*P* < 0.001. ESS, experimental Sjögren’s syndrome; DAPI, 4',6-diamidino-2-phenylindole.

The ESS animal model was established in female wild-type BALB/c mice via subcutaneous injection of emulsified adjuvants and salivary gland autoantigens. The results indicated that body weight (Fig. 3A), salivary secretion (Fig. 3B), and the salivary gland index (Fig. 3C) were significantly decreased in ESS model compared to control groups. In addition, F4/80^+^ macrophage infiltration was evident in the salivary gland (Fig. 3E, F), suggesting activation of the innate immune response. A recent study has suggested that salivary secretion is associated with aquaporin-5 (AQP5), which is downregulated in the salivary glands of pSS patients (13). Compared with the control group, the mRNA of *Aqp5* was downregulated in the salivary gland of ESS mice (Fig. 3D).

Patients with pSS typically exhibit multisystem extraglandular involvement, with the lungs being the most common affected organ. Further analysis revealed that the lung index in ESS model mice was significantly higher than that in control mice (Fig. 3G). H&E staining revealed disrupted alveolar architecture, including pulmonary septal rupture, fusion of destroyed alveolar cavities into emphysematous bullae, and the presence of inflammatory cells in alveolar spaces. The mean linear intercept was significantly greater in the ESS group compared to the control group (Fig. 3H, J). Sirius red staining demonstrated substantial collagen accumulation in the bronchial walls and alveolar septa in ESS mice. Masson’s trichrome staining provided a visual assessment of fibrosis, with collagen appearing blue and muscle fibers red. In ESS mice, marked collagen deposition was observed around the tracheal and capillary basement membranes. Immunohistochemical staining demonstrated significant upregulation of FN expression in the lungs (Fig. 3I, J). Full original animal data have been included in Supplementary Table S1. In summary, the ESS mice model was successfully established, exhibiting pathological features associated with pulmonary fibrosis.

### Sphingolipid metabolism is dysregulated in the salivary glands and lungs of ESS mice model and Sa was identified as the key metabolite

**Fig. 4:**
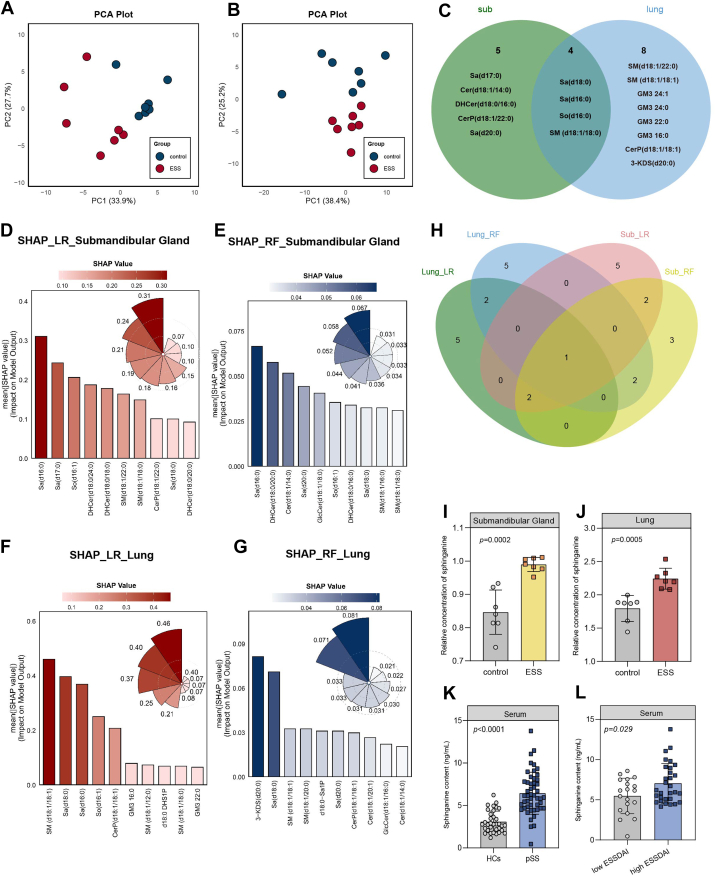
Metabolomic profiling and feature selection in salivary gland and lung tissues of ESS mice. A and B: PCA of sphingolipid profiles in the salivary gland (A) and lung (B), demonstrating distinct separation between control and ESS groups. C: Venn diagram illustrating the distribution of significantly altered sphingolipid metabolites in submandibular gland (green) and lung (blue) tissues. D–G: SHAP value analysis of sphingolipid metabolites in the salivary gland and lung tissues using LR (D and F) and RF (E and G) models. Bar plots show the top 10 metabolites ranked by mean SHAP values; corresponding rose plots depict SHAP value distributions across individual samples. H: Venn diagram showing the overlapping metabolites among the top 10 features identified by LR and RF models in both tissues. I, J: Relative concentrations of Sa in the salivary gland (I) and lung tissues (J) of control and ESS groups. K: Comparison of serum Sa levels between HCs and patients pSS. L: Serum Sa levels in pSS patients stratified by disease activity based on the ESSDAI score. Data are presented as mean ± SD. *P* values were calculated using Student’s *t-*test. pSS, primary Sjögren’s syndrome; ESS, experimental SS; SHAP, Shapley additive explanations; LR, logistic regression; RF, random forest; HC, healthy control; PCA, principal component analysis; ESSDAI, EULAR Sjögren’s Syndrome Disease Activity Index.

Sphingolipid metabolic dysregulation has been increasingly implicated in pSS, with several studies reporting altered levels of ceramides (Cers), Sa, and related metabolites in patient serum and feces (14). Beyond autoimmune disease, sphingolipids have also been linked to pulmonary fibrosis, where Cer accumulation promotes fibroblast activation and collagen deposition, and sphingosine-1-phosphate (S1P) signaling drives profibrotic responses (15, 16). These findings collectively justify the focus on sphingolipid metabolism as a potential shared pathway underlying both pSS and its pulmonary involvement. Therefore, in this study, salivary gland and lung tissues were collected for pseudotargeted sphingolipidomic analysis. Using MRM mode, 12 sphingolipid subclasses were detected, including S1P, sphingosine (So), dihydrosphingosine-1-phosphate (Sa1P), Sa, Cer, ceramide-1-phosphate (Cer1P), hexosylceramide (HexCer), lactosylceramide (LacCer), glucosylceramide (GlcCer), dihydroceramide (DHCer), gangliosides (GM), and sphingomyelin (SM), covering a total of 165 sphingolipid metabolites. A total of 50 sphingolipid metabolites were detected in salivary glands and 79 in lung tissues (Supplementary Table S4, 5). Supplementary Figure S1A and B present the quality control results for salivary gland and lung metabolomic samples, respectively. All quality control samples being within two standard deviations and the 95% confidence interval indicates that the analytical methods were both reliable and acceptable. Unsupervised principal component analysis revealed clear separation between the ESS and control group both in the salivary gland (Fig. 4A) and lung tissue (Fig. 4B). To further identify differential sphingolipid metabolites between the two groups, we applied a screening threshold of *P* < 0.05, which identified 9 and 12 differential metabolites in the salivary gland and lung tissues, respectively. Intersection analysis identified Sa(d18:0), Sa(d16:0), So(d16:0), and SM(d18:1/18:0) as the commonly altered sphingolipid metabolites in both tissues (Fig. 4C).

To improve the accuracy and biological interpretability of metabolic feature selection, machine learning has been widely applied to high-dimensional omics data analysis in recent years (17). Accordingly, LR and RF were employed as complementary machine learning algorithms, and SHAP was used to quantify the contribution and interpretability of each sphingolipid metabolite in model prediction. Figure 4D and E present the SHAP value rankings of sphingolipid metabolites in salivary gland tissues based on LR and RF models, respectively. In both models, Sa(d16:0) ranked as the most influential metabolite contributing to model prediction. Likewise, in lung tissues (Fig. 4F and G), Sa(d18:0) was consistently identified as the second most influential metabolite both in the LR and RF models, highlighting its notable contribution to predictive performance. By integrating the top ten metabolites from both submandibular gland and lung tissues across the two models, Sa was identified as the common key metabolite (Fig. 4H). Sa was consistently elevated both in the salivary gland and lung tissues (Fig. 4I, J) and contributed significantly to classification performance, supporting its potential as a key metabolic biomarker for pSS-related lung injury.

### Validation of elevated Sa levels in serum samples from pSS patients

To further investigate the change trends of Sa in pSS, a total of 83 serum samples were collected, including 34 HCs and 49 pSS patients. No significant differences were found in sex distribution (*P* = 0.631, Chi-square test) or age (*P* = 0.095, independent *t*-test), suggesting demographic similarity between the two groups. Using a targeted metabolomics approach, accurate quantification revealed that the concentration of Sa was 6.4 ± 2.5 ng/ml in pSS patients and 3.1 ± 1.3 ng/ml in the HCs group. The Sa levels were significantly elevated (*P* = 7.336E-12) in the serum of pSS patients compared to HCs (Fig. 4K). Based on the EULAR Sjögren’s Syndrome Disease Activity Index (ESSDAI), which evaluates 12 organ systems, pSS patients were classified into high-activity (ESSDAI≥4, n = 30) and low-activity (ESSDAI<4, n = 19) groups. Notably, Sa levels were significantly higher in the high-activity group, suggesting a potential association between Sa and disease activity (Fig. 4L).

### Sa-induced SS-like symptoms and lung injury in ESS mice

**Fig. 5:**
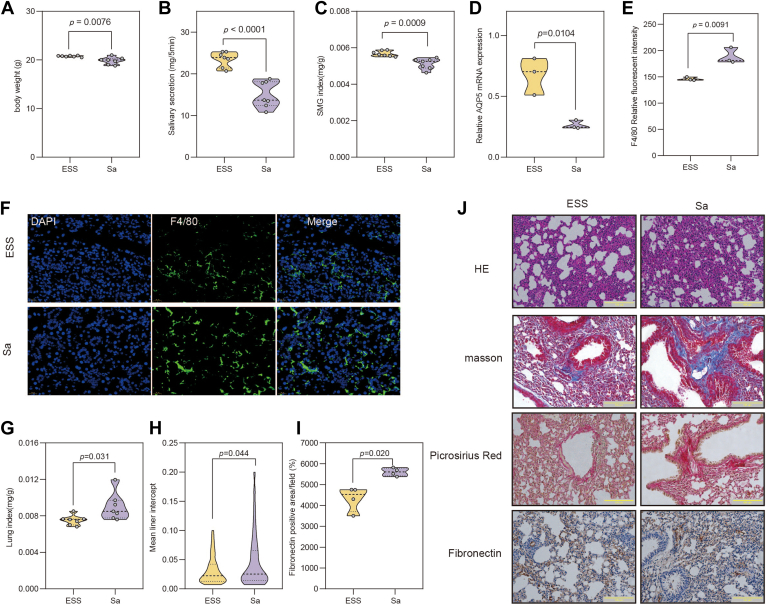
In vivo effects of Sa on salivary gland dysfunction and pulmonary fibrosis in ESS mice. A–C: Comparison of body weight (A), salivary flow rate (B), and submandibular gland index (C) between ESS and Sa-treated groups. D: Relative mRNA expression of *Aqp5* in submandibular gland tissues as measured by quantitative real-time PCR (qRT-PCR). E: Quantification of F4/80^+^ macrophage infiltration in the lung based on relative fluorescence intensity; (F) Immunofluorescence staining of submandibular gland tissues showing F4/80^+^ macrophage infiltration (green); nuclei were counterstained with DAPI (blue). G: Lung index was calculated. Lung index = lung weight (g)/body weight (g) × 100%. H: The mean linear intercept of lung tissues comparisons between ESS and Sa-treated groups. I: Quantification of fibronectin-positive areas in lung tissues. J: Representative histological staining of lung tissues from ESS and Sa-treated mice: H&E staining, Masson’s trichrome staining, Picrosirius red staining for collagen deposition, and immunohistochemical staining of fibronectin. Data are presented as mean ± SD. *P* values were calculated using Student’s *t* test. ESS, experimental SS; DAPI, 4',6-diamidino-2-phenylindole; H&E, hematoxylin and eosin.

To further investigate the role of Sa in ESS mice, Sa was administered by intraperitoneal injection for 14 consecutive days. Compared with the ESS model group, Sa-treated mice exhibited significant reductions in body weight (Fig. 5A), salivary flow rate (Fig. 5B), and salivary gland index (Fig. 5C), suggesting that Sa may further aggravate salivary gland atrophy and impair salivary secretion. In addition, Sa further significantly suppressed *Aqp5* mRNA expression in salivary glands (Fig. 5D), indicating impairment of aquaporin function. Immunofluorescence analysis revealed that Sa treatment further increased F4/80^+^ macrophage infiltration in the salivary glands (Fig. 5E, F). Collectively, these results indicate that Sa may induce the salivary gland dysfunction by simultaneously suppressing aquaporin expression and triggering innate immune activation.

In addition, the effect of Sa administration on the lung tissue was investigated. The lung index was significantly increased in Sa-treated mice compared with the ESS model group (Fig. 5F). H&E staining revealed that Sa treatment further exacerbated inflammatory cell infiltration and induced marked thickening of the alveolar septa in lung tissue (Fig. 5G, J). Masson’s trichrome staining and Sirius red staining further revealed extensive collagen deposition within alveolar septa and interstitial regions in Sa-treated mice, with a markedly greater degree of fibrosis than that observed in the ESS group (Fig. 5J). Immunohistochemical staining additionally showed a significant increase in FN expression compared with the ESS group (*P* < 0.001), supporting the profibrotic role of Sa (Fig. 5I). Overall, these findings demonstrate that Sa exacerbates pulmonary injury associated with pSS.

### Sa induces salivary gland cells (A253 cell) damage via ER stress

**Fig. 6:**
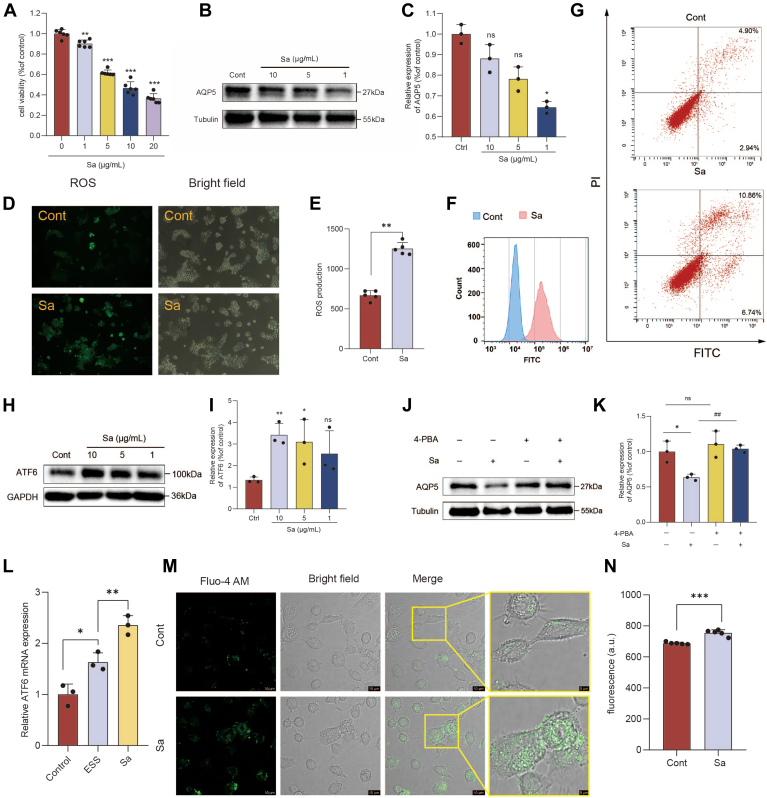
Sa induces A253 cell injury through activation of ER stress. A: Cell viability of A253 cells treated with increasing concentrations of Sa (1–20 μg/ml), assessed by the CCK-8 assay. B and C: Western blot (B) and corresponding quantitative analysis (C) of AQP5 protein levels following Sa treatment. D–F: Intracellular reactive oxygen species (ROS) levels detected by fluorescence microscopy (D), microplate reader (E), and flow cytometry (F). G: Representative FITC/propidium iodide (PI) dual-staining scatter plots showing apoptosis in control and Sa-treated A253 cells. H and I: Western blot (H) and quantitative analysis (I) indicating Sa-induced upregulation of ATF6 protein expression. J and K: Western blot (J) and quantitative analysis (K) demonstrating that ER stress inhibitor 4-PBA reverses Sa-induced AQP5 downregulation. L: Relative mRNA expression of *Atf6* in submandibular gland tissues as measured by qRT-PCR. M and N: Intracellular calcium levels detected by laser confocal microscopy (M), microplate reader (N). Data are presented as mean ± SD. *P* < 0.05, P < 0.01, *P* < 0.001 versus control; ##*P* < 0.01 Sa group with 4-PBA treatment. ER, endoplasmic reticulum; 4-PBA, 4-phenylbutyric acid; CCK-8, Cell Counting Kit-8; PI, propidium iodide; AQP5, aquaporin-5

A253 cells are generally used as an in vitro model to study saliva secretory functions (18). To detect whether Sa can directly cause A253 cell damage, (3-[4,5-dimethylthiazol-2-yl]-2,5 diphenyl tetrazolium bromide) (MTT) assay was used to determine the effect of Sa on the viability of A253 cell. It was observed that Sa could lead to A253 cell damage, with the extent of injury being dose-dependent. (Fig. 6A). Sa treatment led to dose-dependent downregulation of AQP5 expression in A253 cells (Fig. 6B, C). To investigate whether reducing endogenous Sa affects epithelial function, we silenced KDSR in A253 cells. Quantitative real-time PCR (qRT-PCR) and western blot confirmed efficient knockdown of KDSR (Supplementary Fig. S3A, C). Previous studies have shown that loss of KDSR activity leads to a reduction in Sa, highlighting that KDSR is required for its biosynthesis (19). AQP5 expression showed no significant change after KDSR knockdown, suggesting that decreased Sa synthesis does not directly induce epithelial injury (Supplementary Fig. S3G, H). Reactive oxygen species (ROS) plays a crucial role for the cell growth, and the excessive ROS production induces cell apoptosis (20). Figure 6D–F showed that the ROS production in Sa treated group was higher than that in the control group. Moreover, Annexin V-FITC/propidium iodide (PI) double staining was utilized by flow cytometry to distinguish between viable and apoptotic cells. Compared with the control group, the proportion of early apoptotic cells treated with Sa increased from 2.25% to 5.08% and that of late apoptotic cells from 2.94% to 7.16% (Fig. 6G). The results showed significant increase in cell apoptosis after Sa treatment.

The ER, as the primary site of sphingolipid biosynthesis, is likely involved in Sa-induced cellular injury. Previous studies have shown that KDSR, an upstream enzyme in Sa biosynthesis, induces ER stress, and that Sa directly activates the unfolded protein response via ATF6, thereby amplifying ER stress signaling (21, 22). In the submandibular gland, *Atf6* mRNA levels were significantly increased in ESS mice and further elevated after Sa administration (Fig. 6L). Western blot analysis confirmed that Sa significantly upregulated ATF6 expression in A253 cells (Fig. 6H, I). We next examined whether reducing endogenous Sa affects ER stress signaling. Under unstimulated conditions, silencing KDSR did not change ATF6 expression in A253 cells. This indicates that depletion of Sa is insufficient to activate ER stress (Supplementary Fig. S4A, C). Intracellular calcium elevation is well known to interfere with ER function and initiate ER stress. To examine the intracellular calcium levels following Sa treatment, we utilized confocal laser scanning microscopy to visualize intracellular calcium levels in A253 cells by the Fluo-4 AM probe. A notable increase in calcium fluorescence intensity was detected in Sa-treated cells compared to the control group (Fig. 6M). This elevation in intracellular calcium levels was corroborated by a quantification assay conducted using a microplate reader (Fig. 6N). To further assess the role of ER stress in Sa-induced injury in A253 cells, the ER stress inhibitor 4-PBA was administered. Treatment with 4-PBA (5 mM) reversed the Sa-induced downregulation of AQP5 expression (Fig. 6J, K), indicating that ER stress contributes to A253 cell damage. Collectively, these findings indicate that Sa impairs salivary gland function through multiple mechanisms, including the inhibition of proliferation, downregulation of AQP5 expression, increased ROS production, induction of apoptosis, and activation of ER stress in A253 cells.

### Sa promotes fibrotic activation of NIH3T3 fibroblasts via ER stress

**Fig. 7:**
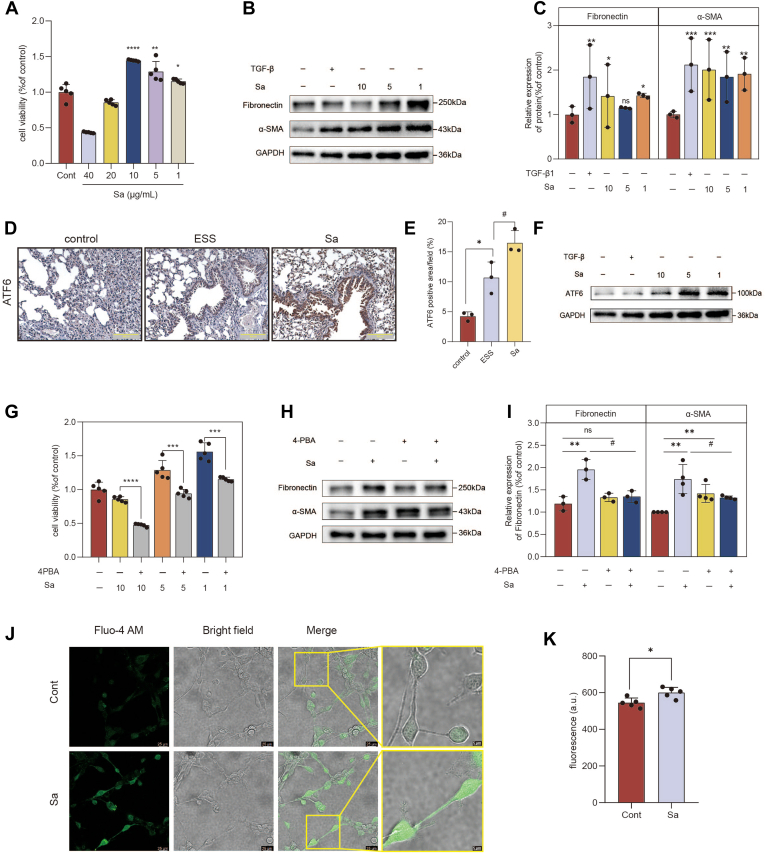
Sa promotes activation and proliferation of NIH3T3 cells through ER stress. A: Cell viability of NIH3T3 cells treated with increasing concentrations of Sa (1–40 μg/ml), assessed by the CCK-8 assay. B and C: Western blot (B) and quantitative analysis (C) of fibronectin and α-SMA expression in NIH3T3 cells treated with varying concentrations of Sa or TGF-β1 (transforming growth factor-beta) (positive control). D: Immunohistochemical staining analysis and (E) quantitative analysis of the expression of ATF6 in lung tissues. F: Western blot analysis of ATF6 expression in NIH3T3 cells following treatment with Sa and/or TGF-β1. G: Cell viability of NIH3T3 cells cotreated with Sa and the ER stress inhibitor 4-PBA (5 mM), measured by CCK-8 assay. H and I: Western blot (H) and corresponding quantification (I) of fibronectin and α-SMA expression in NIH3T3 cells treated with Sa with or without 4-PBA. J and K: Intracellular calcium levels detected by laser confocal microscopy (J), microplate reader (K). Data are presented as mean ± SD. ∗*P* < 0.05, ∗ ∗*P* < 0.01, ∗ ∗ ∗*P* < 0.001, versus control group; #*P* < 0.05 Sa group with 4-PBA treatment; ns, not significant. ER, endoplasmic reticulum; α-SMA, alpha-smooth muscle actin; 4-PBA, 4-phenylbutyric acid; CCK-8, Cell Counting Kit-8

NIH3T3 fibroblasts, a well-established in vitro model for pulmonary fibrosis, were utilized to evaluate the profibrotic effects of Sa. Fibroblast hyperproliferation is recognized as a key hallmark of pulmonary fibrosis (23). As shown in Fig. 7A, Sa promoted NIH3T3 cell proliferation in a dose-dependent manner (1–10 μg/ml). During pulmonary fibrosis, fibroblast activation results in differentiation into alpha-smooth muscle actin (α-SMA) expressing myofibroblasts, accompanied by excessive FN production (24). Sa markedly increased the protein expression levels of FN and α-SMA (Fig. 7B, C). KDSR expression was also successfully reduced in NIH3T3 fibroblasts (Supplementary Fig. S3D–F). Knockdown of KDSR did not affect the expression of FN or α-SMA, indicating that the inhibition of Sa synthesis does not activate fibroblasts or promote a fibrotic phenotype (Supplementary Fig. S3I, J). In the lung tissue, ATF6 expression was markedly upregulated in ESS mice compared with controls, and Sa treatment further enhanced this increase, as shown by immunohistochemistry (Fig. 7D, E). Concurrently, ATF6 expression was detected in NIH3T3 cells, and Sa significantly upregulated its expression (Fig. 7F).

However, KDSR knockdown had no effect on proliferation in ATF6 expression. (Supplementary Fig. S4B, D). Intracellular calcium levels in NIH3T3 cells were measured using the Fluo-4 AM probe. A significant increase in calcium fluorescence was observed following Sa treatment (Fig. 7H, I). Treatment with the ER stress-specific inhibitor 4-PBA (5 mM) completely abolished Sa-induced cell proliferation (Fig. 7G) and reversed the upregulation of FN and α-SMA (Fig. 7H, I). Collectively, these findings demonstrate that Sa promotes fibroblast activation and contributes to pulmonary fibrosis in NIH3T3 cells via an ER stress-dependent mechanism.

### Sa directly binds to Myh9

**Fig. 8:**
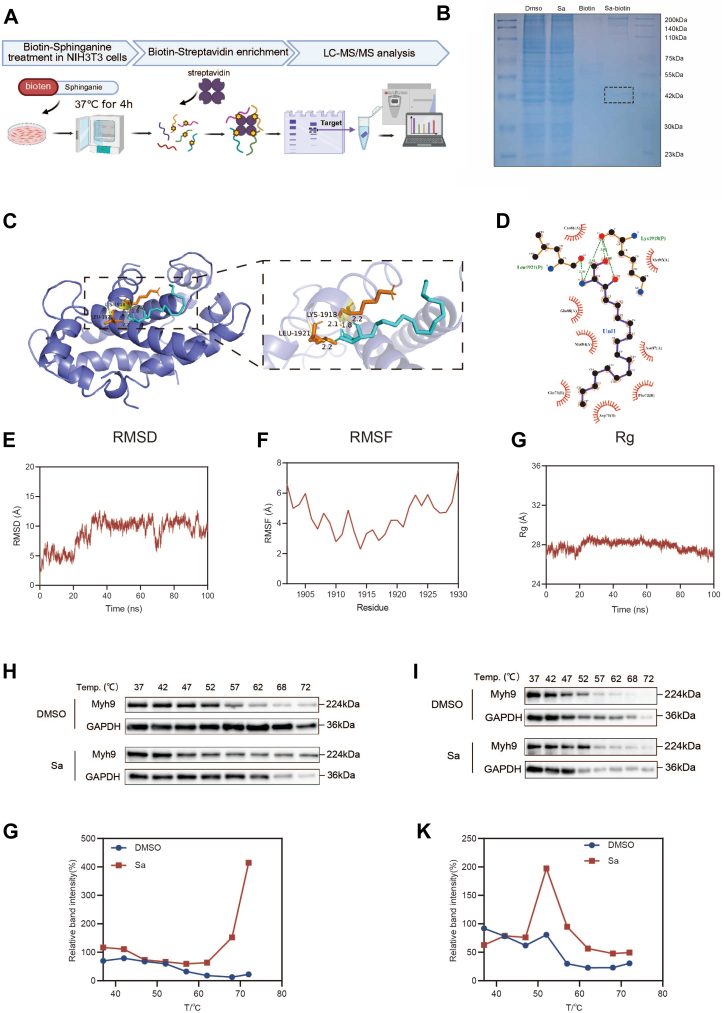
Identification of Myh9 as a direct binding target of Sa and evaluation of complex stability. A: Schematic representation of the chemoproteomic workflow using a biotin-labeled Sa probe in NIH3T3 cells. B: Coomassie blue-stained SDS-PAGE gel showing differential protein bands between treatment groups. The band marked by a dashed box was excised for LC-MS/MS analysis. C: Molecular docking model displaying the binding conformation of Sa with Myh9 and key interacting residues. D: Two-dimensional interaction diagram illustrating hydrogen bonds and hydrophobic interactions between Sa and Myh9. E–G: Molecular dynamics simulation of the Sa–Myh9 complex: (E) RMSD, (F) RMSF of chain D, and (G) Rg plots, reflecting structural stability, residue flexibility, and compactness over 100 ns. H–K: CETSA evaluating Myh9 thermal stability in A253 (H and J) and NIH3T3 (I and K) cells treated with Sa (10 μg/ml). Melting curves derived from CETSA illustrate increased thermal stability of Myh9. Data are presented as mean ± SD. *P* < 0.05, *P* < 0.01 versus control group. CETSA, cellular thermal shift assay; RMSD, root mean square deviation; RMSF, root mean square fluctuation; Rg, radius of gyration.

In situ target identification was conducted by incubating NIH3T3 cells with a biotinylated Sa probe (Sa-biotin) for 4 h. After incubation, cells were lysed, and the probe-bound proteins were enriched using streptavidin magnetic beads. After enrichment, the proteins underwent separation by SDS-PAGE and were identified with LC-MS/MS (Fig. 8A). Coomassie blue staining revealed a distinct band at approximately 40 kDa in the Sa-biotin group, which was excised for mass spectrometry analysis (Fig. 8B). A protein with the highest number of unique peptides in the Sa-biotin group was identified as Myh9.

To further characterize the interaction between Sa and Myh9, molecular docking and molecular dynamics simulations were performed. Docking analysis indicated that Sa primarily binds with Myh9 through hydrogen bonds at Leu1921 and Lys1918, with a binding energy of −5.51 kcal/mol (Fig. 8C). A two-dimensional (2D) interaction diagram revealed additional hydrophobic interactions between Sa and several surrounding residues, including Met84, Met85, Glu88, Asn87, Gln73, Phe72, and Asp71, thereby stabilizing the Sa-Myh9 complex (Fig. 8D).

The structural stability and dynamic properties of the Sa–Myh9 complex were evaluated through a 100-ns MD simulation. Root mean square deviation analysis demonstrated that the complex reached equilibrium after ∼30 ns, indicating structural stability over the entire simulation period (Fig. 8E). Root mean square fluctuation (RMSF) analysis was performed to assess residue-level flexibility. Due to the size and complexity of Myh9, the protein was divided into four chains (A–D) for separate analysis. The RMSF analysis indicated that chains A–C demonstrated greater overall flexibility, characterized by several regions exhibiting peak fluctuations exceeding 7 Å (Supplementary Fig. S6A–C). Conversely, chain D exhibited markedly reduced flexibility, with RMSF values primarily between 2.0 Å and 5.5 Å. A particularly notable decrease in flexibility was observed in the 1910–1925 residue range, where the minimum fluctuation was as low as 2.1 Å. This region corresponds to the predicted binding site identified through molecular docking, implying that Sa binds more stably to chain D (Fig. 8F). The radius of gyration (Rg) remained consistently around 28 Å with minimal fluctuation throughout the simulation, suggesting a compact and stable complex structure (Fig. 8G). Overall, Sa stably interacts with Myh9 while maintaining both global and local conformational stability.

The direct interaction between Sa and Myh9 was validated using the cellular thermal shift assay in both A253 and NIH3T3 cells. In A253 cells, Myh9 protein levels declined rapidly with increasing temperatures in the control group, whereas Sa treatment preserved higher Myh9 levels at higher temperatures, leading to a rightward shift in the melting curve and enhanced thermal stability (Fig. 8H, G). Similar results were observed in NIH3T3 cells, in which Sa treatment maintained Myh9 stability at intermediate-to-high temperatures (approximately 47–57 °C), thereby demonstrating its role in enhancing Myh9 thermal stability (Fig. 8I, K). Collectively, these findings confirm that Sa enhances the thermal stability of Myh9 through direct binding.

### Sa induces epithelial injury in A253 cells and fibroblast activation in NIH3T3 cells via Myh9 upregulation

**Fig. 9:**
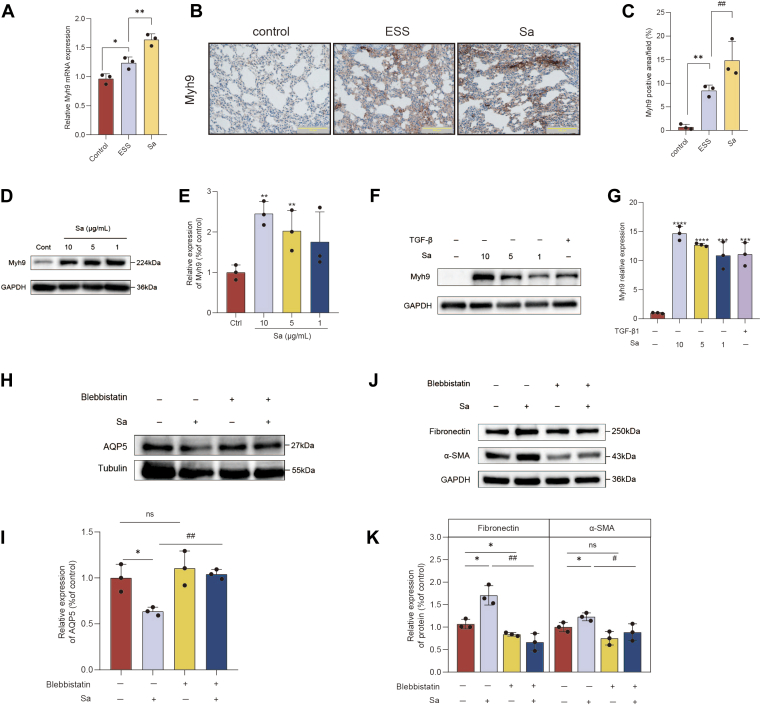
Functional role of Myh9 in Sa-induced epithelial injury and fibroblast activation. A: Relative mRNA expression of Myh9 in submandibular gland tissues as measured by qRT-PCR. B: Immunohistochemical staining analysis and (C) quantitative analysis of the expression of Myh9 in lung tissues. D and E: Western blot analysis (D) and corresponding quantification (E) of Myh9 protein expression in A253 cells treated with increasing concentrations of Sa. F and G: Western blot (F) and densitometric analysis (G) of Myh9 expression in NIH3T3 cells treated with Sa or TGF-β1 (positive control). H and I: Western blot (H) and quantification (I) of AQP5 expression in A253 cells treated with Sa, with or without the Myh9 inhibitor blebbistatin. J and K: Western blot (J) and quantification (K) of fibronectin and α-SMA protein levels in NIH3T3 cells treated with Sa, with or without blebbistatin. Data are presented as mean ± SD. ∗*P* < 0.05, ∗∗*P* < 0.01, ∗∗∗*P* < 0.001, ∗∗∗∗*P* < 0.0001; ns, not significant. #*P* < 0.05, ##*P* < 0.01 Sa group with 4-PBA treatment. α-SMA, alpha-smooth muscle actin; 4-PBA, 4-phenylbutyric acid; AQP5, aquaporin-5

To further clarify the role of Myh9 in Sa-induced injury, we first examined its expression in target organs. In the submandibular gland, Myh9 mRNA levels were significantly increased in ESS mice and further elevated after Sa administration (Fig. 9A). Consistently, immunohistochemistry of lung tissue demonstrated markedly enhanced Myh9-positive staining in the ESS group, which was further increased in Sa-treated mice (Fig. 9B–C), indicating that Myh9 is upregulated in both salivary gland and lung during disease progression. After confirming that Sa directly binds to and stabilizes Myh9, the functional role of Myh9 in epithelial injury in A253 cells and fibroblast activation in NIH3T3 cells was further investigated. Sa treatment markedly increased Myh9 (Fig. 9D, E) and decreased AQP5 (Fig. 6B, C) protein expression in A253 cells. To further elucidate the role of Myh9, A253 cells were treated with blebbistatin (a Myh9-specific inhibitor, 2 μM). Notably, blebbistatin reversed the Sa-induced reduction of AQP5 protein expression, suggesting that Myh9 is involved in Sa-mediated epithelial dysfunction in the salivary gland (Fig. 9H, I). In NIH3T3 cells, Sa also induced the upregulation of Myh9, FN, and α-SMA protein (Fig. 9F, G). Conversely, blebbistatin blocked the upregulation of fibrosis markers FN and α-SMA induced by Sa (Fig. 9J, K), while supporting the involvement of Myh9 in fibroblast activation. Western blot analysis showed that KDSR silencing did not alter Myh9 protein expression in either A253 epithelial cells or NIH3T3 fibroblasts (Supplementary Fig. S5). Collectively, these findings identify Myh9 as a key mediator of Sa-induced salivary gland epithelial dysfunction and fibroblast activation, thereby contributing to lung involvement in the pathogenesis of pSS.

## Discussion

Pulmonary complications are the most common extraglandular manifestations of pSS, with prevalence estimates ranging from approximately 8%–38%. Despite this, the pathogenic mechanisms responsible for pulmonary involvement in pSS are not yet fully elucidated. It is generally accepted that both glandular and extraglandular manifestations in pSS originate from similar pathogenic processes, such as epithelial lymphocytic infiltration and immune-mediated tissue injury, although the exact mechanisms remain undefined. Dysregulation of lipid metabolism has been identified as a critical factor in the pathogenesis of pSS, with alterations in exocrine gland lipids being closely linked to local inflammatory responses and potentially serving as early risk factors for autoimmune tissue injury (25). Current research on lipid metabolism in pSS has predominantly focused on circulating lipoproteins (26), fatty acids (27), and specific proinflammatory or proresolving lipid mediators (28) implicated in disease progression. S1P and its receptor S1PR have been shown to modulate the autoimmune phenotype of pSS through their actions on both immune and epithelial cells (29). Prior research has demonstrated that sphingolipid metabolism is crucial in the advancement of lung fibrosis (30). Hence, sphingolipid metabolism may serve as a common regulatory pathway involved in pulmonary involvement associated with pSS.

Functional metabolomics, also known as “active metabolomics”, has gained traction as a method to explore the roles of metabolites in maintaining both physiological and pathological homeostasis (31). Although numerous studies have identified metabolite-based diagnostic biomarkers for pSS, the mechanistic roles of these differential metabolites in the pathogenesis of pSS remain underexplored. In this study, SHAP analysis combined with *P* value filtering identified significant elevation of Sa in both the salivary glands and lung tissues of the ESS model mice. Previous research has demonstrated that Sa levels are markedly increased in the serum of patients with ventilator-associated lung injury and are highly expressed in the bronchoalveolar lavage fluid of individuals with cystic fibrosis (32, 33).

In the present study, we demonstrated that Sa was elevated in pSS patients, and this elevation was associated with ESSDAI. Sa enhances TLR4 signaling via MyD88 recruitment, both of which are upregulated in pSS labial glands and associated epithelial and immune cells (34, 35).

In this study, exogenous Sa administration led to a tissue-specific remodeling of sphingolipid metabolism. In the submandibular gland, levels of Sa, dhCer, and Cer were decreased rather than increased after treatment. In contrast, downstream complex sphingolipids—including SM, GlcCer, and LacCer—were significantly elevated (Supplementary Fig. S7A). This metabolic pattern suggests that Sa is rapidly consumed and diverted toward the synthesis of complex sphingolipids rather than accumulating in its free or intermediate forms. Notably, these complex sphingolipids have been reported to promote arachidonic acid release and proinflammatory mediator production, thereby exacerbating salivary gland injury (36, 37). Unlike in the salivary gland, Sa supplementation in the lung increased upstream Sa levels but resulted in a decrease in dhCer, Cer, and most downstream sphingolipids (Supplementary Fig. S7B). It implies an interrupted metabolic flow and further confirms in vivo Sa accumulation. This phenomenon may be attributed to ER stress–mediated suppression of sphingolipid biosynthesis or to accelerated lipid turnover, ultimately leading to a net decrease in downstream metabolites. Accordingly, Sa was selected as a key metabolite for further validation to elucidate its potential role in pSS pathogenesis.

Sphingolipid synthesis has been documented to be associated with the ER, where ER stress may further augment Sa production (38). Notably, Sa has been demonstrated to interact with the transmembrane region of ATF6, thereby initiating downstream signaling pathways (22). Furthermore, dysregulated activation of the ATF6α-associated unfolded protein response pathway has been observed in the salivary glands of patients with pSS and is strongly correlated with elevated expression of proinflammatory cytokines (39). In the present study, Sa was found to significantly upregulate ATF6 expression in salivary gland epithelial cells, and its role in epithelial cell injury was further corroborated by the activation of the ER stress pathway. Importantly, ER stress is also a pivotal factor in the pathogenesis of pulmonary fibrosis. Persistent ER stress has been implicated in promoting epithelial damage and driving epithelial–mesenchymal transition, in addition to activating fibrogenic pathways such as transforming growth factor-β (TGF-β)/Smad signaling, which ultimately contributes to fibroblast activation and excessive collagen accumulation (40). Collectively, these findings suggest that Sa-induced ER stress may serve as a common mechanistic pathway contributing to both submandibular gland injury and pulmonary fibrosis, thereby offering novel insights and therapeutic opportunities for addressing multiorgan involvement in pSS.

Myh9, a constituent of the myosin heavy chain (MyHC) family, encodes nonmuscle myosin IIA, which plays a crucial role in cellular migration, contraction, development, morphological maintenance, and adhesion (41). Previous studies have demonstrated that Myh9 expression is elevated in both bleomycin-induced murine models and TGF-β1-treated MRC-5 human lung fibroblasts, thereby contributing to fibrosis through the activation of the ALK5/Smad2/3 signaling pathway (42). Additionally, TP53TG1 has been identified as interacting with Myh9, leading to the downregulation of its expression, inhibition of fibroblast activation, and mitigation of pulmonary fibrosis (43). Although a direct link between Myh9 and pSS has not been established, clinical observations indicate that thrombocytopenia affects 5%–16% of pSS patients and is inversely related to the severity of pulmonary involvement (44). Myh9 is known to regulate megakaryocyte migration, chemotaxis, and cytokinesis, and its dysregulation may lead to impaired platelet production or function (45). In the present study, Sa was observed to bind directly to Myh9, enhancing its thermal stability and upregulating its expression in both submandibular gland epithelial cells and fibroblasts. The persistent upregulation of Myh9 indicates its potential involvement in pulmonary fibrosis associated with pSS, possibly through the dysregulation of thrombopoiesis and platelet function. This finding offers novel molecular insights into the multi-organ pathogenesis of pSS.

Previous studies have demonstrated that intracellular calcium levels not only serve as key regulators of Myh9 expression but also participate in calcium signaling processes. On one hand, elevated calcium concentrations can significantly activate the myosin heavy chain promoter, thereby markedly increasing Myh9 expression (46). On the other hand, Myh9 itself is involved in modulating calcium signaling, as its knockdown has been shown to impair antigen-induced calcium responses (47). In our study, treatment with Sa led to a notable increase in intracellular calcium levels, accompanied by upregulation of Myh9 expression. Calcium influx is also known to trigger ER stress, and we observed a concomitant increase in the expression of the ER stress-related transcription factor ATF6 (48).

In addition to these mechanistic findings, the pharmacokinetic behavior of Sa after intraperitoneal administration warrants consideration. Safingol—the L-threo stereoisomer of Sa undergo rapid metabolic conversion and broadly distribute into the circulation and peripheral organs, which is consistent with our pseudotargeted metabolomics data showing a rapid increase of Sa in both the lung and submandibular gland after administration (49). Prior toxicokinetic studies of safingol have similarly demonstrated systemic absorption and wide tissue distribution, and Phase I clinical data indicate tolerance at doses up to 840 mg/m^2^ (≈280 mg/kg in mice), supporting the safety margin of the 20 mg/kg dose used here (50). Beyond its distribution, safingol, a Sa analog, can inhibit SPHK1 and reduce S1P production, suggesting that transiently elevated plasma levels of Sa may further perturb the S1P–Treg regulatory axis that maintains immune homeostasis in pSS (51). Given these uncertainties, we acknowledge that the present work does not fully characterize Sa pharmacokinetics in vivo. Future studies using isotope-labeled tracers and optimized delivery strategies will be needed to clarify exposure profiles and determine whether lower or more controlled dosing regimens can achieve comparable biological effects.

Although the ESS mouse model reproduces key features of pSS, including reduced salivary flow, acinar epithelial injury, immune cell infiltration, and partial pulmonary alterations, it does not fully mimic the chronic, heterogeneous, and systemic nature of human disease. In particular, this model is induced by submandibular gland protein immunization rather than spontaneous autoimmunity, and it may not recapitulate long-term immune dysregulation, autoantibody maturation, or the full spectrum of interstitial lung lesions observed in patients. Therefore, future studies using genetic models or spontaneous pSS models will be necessary to validate these findings in vivo.

**Fig. 10:**
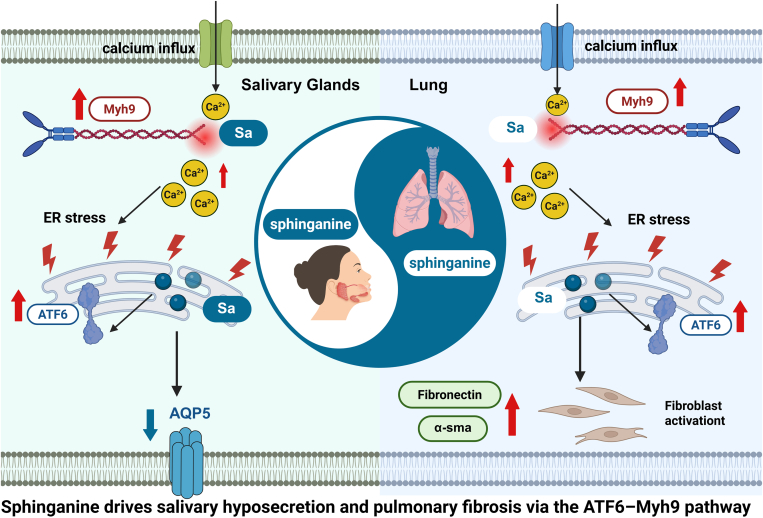
Molecular mechanism of Sa induces salivary hyposecretion and pulmonary fibrosis in pSS through the ATF6–Myh9 signaling pathway. Sa, sphinganine; pSS, primary Sjögren’s syndrome.

In summary, Sa accumulation may contribute to salivary epithelial injury and pulmonary fibroblast activation, potentially via the ATF6–Myh9 pathway. Although this mechanism is supported by in vitro data, the in vivo evidence remains correlative. Thus, Sa should be considered a putative metabolic mediator rather than a confirmed driver of pSS-related pulmonary involvement. The proposed mechanism is shown in Fig. 10 and warrants further validation in genetic or pharmacological models.

## Conclusion

This study introduces an innovative approach for identifying key metabolic biomarkers linked to lung involvement in pSS. A bibliometric analysis highlighted pulmonary involvement as a significant and emerging area of focus in pSS research. Utilizing pseudotargeted sphingolipidomics, the study systematically compared metabolic profiles between target organs (salivary gland) and affected organs (lungs) in a pSS mouse model. This approach led to the identification of Sa as a critical metabolite through machine learning-based feature selection. Functional validation demonstrated that Sa induces epithelial injury in salivary glands and promotes pulmonary fibrosis, primarily through the activation of the ER stress pathway. Furthermore, chemoproteomic profiling identified Myh9 as a direct binding target of Sa, suggesting a key role in the development of pulmonary complications in pSS.

## Ethics Approval and Consent to Participate

This study was approved by the ethics committee of the Second Hospital of Shanxi Medical University (2022YX063), and all participants gave written informed consent.

## Data availability

The datasets used and/or analyzed during the current study are available from the corresponding author on reasonable request.

## Supplemental data

This article contains supplemental data.

## Conflict of interests

The authors declare that they have no conflicts of interest with the contents of this article.
