# Supplementary material for: Sphinganine as a potentially relevant metabolite in pulmonary involvement of primary Sjögren’s syndrome
Source: J Lipid Res. 2025 Dec 11;67(1):100961. doi: 10.1016/j.jlr.2025.100961 (PMC12814850; doi:10.1016/j.jlr.2025.100961)
Supplement: Supplementary Material 1 [file mmc1.docx]

**Supporting Information for**

**Original article**

**Sphinganine as a potentially relevant metabolite in pulmonary involvement of primary Sjögren’s syndrome**

**Authors: Ting Cui^1,4#^, Ziying Geng^2#^, Nan Wang^3,5^, Jing Luo^3,5*^ and Zhenyu Li^1,4*^**

*^1^Modern Research Center for Traditional Chinese Medicine of* *Shanxi University, Taiyuan 030000, China,*

*^2^ School of Pharmacy, Shanxi Medical University, Taiyuan 030000, China.^33^* *Department of Rheumatology, The Second Hospital of Shanxi Medical University, Taiyuan, Shanxi, China.^4^the Key Laboratory of Chemical Biology and Molecular Engineering of Ministry of Education, Shanxi University,* *Taiyuan 030000, China.*

*^5^* *Shanxi Key Laboratory of Rheumatism Immune Microecology, Taiyuan, Shanxi, China.*

#These authors made equal contributions to this work

*Corresponding authors.

**Corresponding authors:** Prof. Zhenyu Li, Modern Research Center for Traditional Chinese Medicine of Shanxi University, No. 92, Wucheng Road, Taiyuan 030006, Shanxi, People’s Republic of China. E-mail: [lizhenyu@sxu.edu.cn](mailto:lizhenyu@sxu.edu.cn)

Prof. Jing Luo, Division of Rheumatology, Department of Medicine, The Second Hospital of Shanxi Medical University, No. 382, Wuyi Road, Taiyuan 030006, Shanxi, People’s Republic of China. E-mail: [ljty966@hotmail.com](mailto:ljty966@hotmail.com)

[**Supplementary methods 2**](#_Toc213247366)

[**Supplementary tables 4**](#_Toc213247367)

[**Supplementary figures 12**](#_Toc213247368)

# Supplementary methods

**Quantitative real-time PCR (qRT-PCR) for relative gene expression**

Total RNA was isolated using a Mei5bio RNA isolation system kit from China, following the manufacturer's instructions, and then transcribed into cDNA using the Monad Biotech cDNA kit. The 2×Q5 SYBR qPCR Mix from ToloBio-technology (Shanghai, China) was used for qPCR analysis. Primers used for gene expression analysis by RT-qPCR were *Aqp5* (5′-CACATCAATCCGGCCATTACTC-3′ and 5′-CGCATTGACGGCCAGGTTA-3′) and *Gapdh* (5′- CCTCGTCCCGTAGACAAAATG-3′ and 5′- TGAGGTCAATGAAGGGGTCGT -3′).

**Western blot analysis**

A253 and NIH-3T3 cells were seeded in 6-well plates (2 × 10⁵ cells/well) and cultured for 24 hours, followed by treatment with sphinganine (0, 1, 5, and 10 μg/mL) for 24 hours. After treatment, cells were lysed with RIPA buffer (Solarbio) containing PMSF, and protein concentrations were measured using a BCA assay. Equal amounts of protein (30 µg) were separated by 8% or 10% SDS-PAGE and transferred to PVDF membranes (Millipore).

Membranes were blocked with 5% non-fat milk for 2 hours at room temperature and incubated overnight at 4°C with primary antibodies: anti-AQP5 (1:1000, Proteintech, China), anti-ATF6 (1:1000, Proteintech, China), anti-FN (1:1000, Proteintech, China), anti-α-SMA (1:1000, Proteintech, China), anti-Myh9 (1:1000, Proteintech, China), anti-GAPDH (1:20000, Bioss, China), anti-Tubulin (1:1000, Solarbio, China), and anti-β-actin (1:20000, Proteintech, China). After washing, membranes were incubated with HRP-conjugated secondary antibodies: goat anti-mouse (1:20000) and goat anti-rabbit (1:20000) for 1.5 hours at room temperature. Protein bands were visualized using ECL substrate and imaged with the ChemiDoc™ XRS+ system (Bio-Rad, Hercules, CA, USA).

**Reactive Oxygen Species and Apoptosis Assay**

The Annexin V-FITC/propidium iodide (PI) apoptosis detection kit and reactive oxygen species (ROS) detection kit were obtained from Servicebio (Wuhan, China).

For the ROS assay, A253 cells seeded into 96-well and 6-well plates were incubated at 37°C with 10 μM dichlorodihydrofluorescein diacetate (DCFH-DA) for 30 minutes. Cells were washed three times with phosphate-buffered saline and then incubated in fresh culture medium containing various treatments. After treatment, absorbance was measured using a fluorescence microplate reader at excitation and emission wavelengths of 485 and 535 nm, respectively. Immunofluorescence microscopy was then used to capture the ROS levels. Finally, the stained cells were analyzed by flow cytometry (Beckman Coulter, Inc., Brea, CA, United States).

The Annexin V-FITC/PI kit was used to detect cell apoptosis by flow cytometry. A253 cells were collected, adjusted to 1×10^6 cells/mL, and stained with Annexin V-FITC and PI solution for 15 minutes at room temperature in the dark. Finally, the stained cells were analyzed by flow cytometry (Beckman Coulter, Inc., Brea, CA, United States).

**Cell calcium imaging with Fluo-4**

Fluo-4 acetoxymethyl ester (Fluo-4-AM) (Beyotime, Beijing, China) dye was used to measure intracellular calcium. Cells were plated on laser confocal dishes at a density of 2× 10^5^ cells/well and treated as described above. After treatment, the cells were stained with Fluo-4-AM diluted to a concentration of 2.5 μM for 30 min in darkness at 37 °C and then washed with Dulbecco’s phosphate-buffered saline (DPBS) three times. The green fluorescence, which reflected the intracellular calcium level, was recorded by a fluorescence microscope (Leica, Wetzlar, Germany).

**Molecular Docking**

Molecular docking was conducted using AutoDock Vina with semi-flexible docking. Protein crystal structures were obtained from the RCSB PDB database (<http://www.rcsb.org/>), selected based on: *Homo sapiens* origin, physiological pH, inclusion of original ligands, and resolution ≤ 2.5 Å. Water molecules and ligands were removed using PyMOL.

3D structures of active compounds were downloaded from PubChem. Receptors and ligands were preprocessed by hydrogenation using AutoDock Tools 1.5.7. The grid box was centered on the original ligand binding site to ensure complete coverage of potential binding pockets. Binding free energies were calculated by AutoDock Vina. Docking results were visualized and analyzed using Discovery Studio 2024, PyMOL 2.5.8, and LigPlot, which highlighted various interactions between ligands and proteins, including electrostatic interactions, hydrogen bonds, and hydrophobic contacts. Binding stability was assessed by the minimum binding energy, with values ≤ -5 kcal/mol considered strong binding.

**Molecular dynamics simulation**

Molecular dynamics (MD) simulations were performed using Gromacs 2022 software for 100 ns. The CHARMM36 force field was applied for proteins, while GAFF2 was used for ligands. The TIP3P water model was used to solvate the protein-ligand complex in a cubic water box with a 1.2 nm periodic boundary. Electrostatic interactions were calculated using the Particle Mesh Ewald (PME) method, and the Verlet algorithm was applied for neighbor searching. Energy minimization was followed by equilibration using NVT and NPT ensembles for 100 ps with a coupling constant of 0.1 ps. Both van der Waals and Coulomb interactions were calculated using a 1.0 nm cutoff. Finally, production MD was run at 310 K and 1 bar for 100 ns.

# Supplementary tables

## Table S1 Raw physiological and pathological indicators of mice across Control, ESS, and Sphinganine-treated groups

|  | group | | |
| --- | --- | --- | --- |
|  | Control | ESS | Sphinganine |
| body weight | 22.44±0.8938 | 20.6156±0.3224 | 19.8429±0.7399 |
| Salivary secretion | 33.5±1.747 | 23.5286±1.8209 | 15.0143±3.1683 |
| SMG index | 0.0064±0.0005 | 0.0057±0.0002 | 0.0051±0.0003 |
| Lung index | 0.0063±0.0003 | 0.0075±0.0005 | 0.009±0.0015 |
| Mean liner intercept | 0.0191±0.0154 | 0.0314±0.0235 | 0.0465±0.0456 |
| Fibronectin positive area/field | 3473.1707±136.4962 | 4579.3265±220.3261 | 5603.9265±192.8797 |
| F4/80 fluoresoent intensity | 104.6527±7.2676 | 145.992±3.2953 | 188.4187±15.1899 |

## Table S2 Serum sample donors' demographic Information

| Group | Sample size | Age (Year ± SD) | Sex (M/F) | ESSDAI(hour ± SD) |
| --- | --- | --- | --- | --- |
| HCs | 34 | 55.6+5.7 | 4/30 |  |
| pSS | 49 | 58.8+9.8 | 3/46 | 6.4+2.5 |

## Table S3 Optimized quantitative MRM transitions of compounds in positive ion mode.

| **identification** | **DP** | **CE** | **Q1** | **Q2** | **Retention time** |
| --- | --- | --- | --- | --- | --- |
| So d20:1 | 70 | 20 | 328.4 | 310.4 | 8.71 |
| So d18:1 | 63 | 26 | 300.3 | 252.3 | 7.61 |
| So d17:1 | 70 | 15 | 286.1 | 268.1 | 9.56 |
| So d16:1 | 80 | 15 | 272.4 | 254.4 | 5.19 |
| SM(d18:1/22:0) | 90 | 45 | 787.7 | 184.3 | 18.16 |
| SM(d18:1/20:0) | 90 | 45 | 759.7 | 184.3 | 16.45 |
| SM (d18:1/18:0) | 60 | 45 | 731.8 | 184.4 | 14.98 |
| SM (d18:1/24:1) | 60 | 40 | 813.7 | 184.1 | 22.40 |
| SM (d18:1/24:0) | 60 | 40 | 815.7 | 184.1 | 23.61 |
| SM (d18:1/18:1) | 60 | 40 | 729.6 | 184.1 | 14.72 |
| SM (d18:1/16:0) | 60 | 40 | 703.5 | 184.1 | 15.74 |
| SM (d18:1/12:0) | 45 | 40 | 647.6 | 184.3 | 13.27 |
| S1P d20:1 | 90 | 24 | 408.4 | 292.4 | 18.96 |
| S1P d20:0 | 110 | 19 | 410.4 | 312.4 | 10.21 |
| S1P d18:1 | 90 | 25 | 380.2 | 264.3 | 15.22 |
| S1P d18:0 | 90 | 19 | 382.4 | 284.4 | 13.02 |
| S1P d17:1 | 80 | 20 | 366.1 | 250 | 0.95 |
| S1P d17:0 | 100 | 17 | 368.4 | 270.4 | 9.10 |
| S1P d16:1 | 90 | 25 | 352.4 | 236.4 | 4.04 |
| S1P d16:0 | 90 | 19 | 354.4 | 256.4 | 11.84 |
| PhytoSo | 60 | 32 | 318.4 | 282.4 | 5.12 |
| LacCer(d18:1/24:1) | 60 | 60 | 972.7 | 264.3 | 21.04 |
| LacCer(d18:1/24:0) | 60 | 60 | 974.8 | 264.3 | 23.99 |
| LacCer(d18:1/22:0) | 60 | 60 | 946.7 | 264.3 | 20.89 |
| LacCer(d18:1/20:0) | 60 | 60 | 918.7 | 264.3 | 18.57 |
| LacCer(d18:1/18:1) | 60 | 60 | 888.7 | 264.3 | 24.40 |
| LacCer(d18:1/18:0) | 60 | 60 | 890.7 | 264.3 | 16.67 |
| LacCer(d18:1/16:0) | 60 | 60 | 862.6 | 264.3 | 15.23 |
| LacCer(d18:0/24:0) | 60 | 55 | 976.7 | 266.4 | 23.99 |
| LacCer(d18:0/16:0) | 60 | 50 | 864.7 | 266.4 | 15.23 |
| LacCer (d18:1/12:0) | 45 | 55 | 806.6 | 264.4 | 23.21 |
| IS:C17:0-Cer | 65 | 35 | 552.3 | 264.5 | 17.21 |
| HexSph 18:1 | 60 | 15 | 462.1 | 282.2 | 12.28 |
| HexCer 8:0 | 40 | 30 | 588.2 | 264.2 | 14.78 |
| HexCer 24:1 | 80 | 36 | 810.2 | 264.1 | 21.75 |
| HexCer 24:0 | 70 | 36 | 812.2 | 264.1 | 24.94 |
| HexCer 22:1 | 80 | 40 | 782.3 | 264.2 | 21.05 |
| HexCer 22:0 | 80 | 39 | 784.3 | 264.2 | 21.64 |
| HexCer 20:1 | 80 | 40 | 754.3 | 264.2 | 18.68 |
| HexCer 20:0 | 80 | 45 | 756.3 | 264.2 | 19.15 |
| HexCer 18:1 | 60 | 35 | 726.1 | 264.2 | 16.81 |
| HexCer 18:0 | 70 | 35 | 728.2 | 264.2 | 17.12 |
| HexCer 16:1 | 60 | 35 | 698.2 | 264.2 | 15.24 |
| HexCer 16:0 | 60 | 37 | 700.2 | 264.2 | 15.50 |
| HexCer 14:1 | 65 | 35 | 670.2 | 264.1 | 19.82 |
| HexCer 14:0 | 75 | 35 | 672.2 | 264.1 | 14.22 |
| HexCer 12:1 | 65 | 30 | 642.2 | 264.1 | 18.18 |
| HexCer 12:0 | 75 | 35 | 644.2 | 264.1 | 19.62 |
| GM3 (d18:1/24:1) | 70 | 60 | 1263.8 | 264.3 | 20.29 |
| GM3 (d18:1/24:0) | 70 | 60 | 1265.8 | 264.3 | 23.01 |
| GM3 (d18:1/22:0) | 70 | 60 | 1237.8 | 264.3 | 20.16 |
| GM3 (d18:1/20:0) | 70 | 60 | 1209.8 | 264.3 | 14.46 |
| GM3 (d18:1/18:1) | 70 | 60 | 1179.8 | 264.3 | 21.38 |
| GM3 (d18:1/18:0) | 70 | 60 | 1181.7 | 264.3 | 16.15 |
| GM3 (d18:1/16:0) | 70 | 60 | 1153.7 | 264.3 | 14.77 |
| GlcCer (d18:1)/C24:1 | 35 | 55 | 810.9 | 264.4 | 21.74 |
| GlcCer (d18:1)/C12:0 | 70 | 42.5 | 644.6 | 264.4 | 19.61 |
| dhSph d20:0 | 90 | 30 | 330.4 | 312.4 | 7.72 |
| dhSph d18:0 | 65 | 30 | 302.1 | 254.3 | 7.94 |
| dhSph d17:1 | 70 | 15 | 288.1 | 270.2 | 10.59 |
| dhSph d17:0 | 50 | 45 | 288.4 | 60 | 2.39 |
| dhSph d16:0 | 80 | 25 | 274.4 | 256.4 | 5.10 |
| DHSM (d18:0)/C24:0 | 45 | 50 | 817.9 | 184.4 | 16.88 |
| DHSM (d18:0)/C18:0 | 45 | 45 | 733.8 | 184.4 | 15.96 |
| DHSM (d18:0)/C12:0 | 45 | 40 | 649.7 | 184.4 | 13.44 |
| DHGlcCer (d18:0)/C24:0 | 35 | 55 | 814.9 | 266.4 | 24.94 |
| DHGlcCer (d18:0)/C18:0 | 35 | 47.5 | 730.7 | 266.4 | 21.19 |
| DHGlcCer (d18:0)/C16:0 | 35 | 45 | 702.7 | 266.4 | 18.86 |
| DHGlcCer (d18:0)/C12:0 | 35 | 42.5 | 646.6 | 266.4 | 21.39 |
| DHCer1P (d18:0)/C24:0 | 60 | 25 | 732.9 | 266.4 | 21.24 |
| DHCer1P (d18:0)/C16:0 | 40 | 47.5 | 564.6 | 266.4 | 10.18 |
| DHCer(d18:0/15:0) | 55 | 37.5 | 526.6 | 266.4 | 9.88 |
| DHCer(d18:0/14:0) | 55 | 35 | 512.6 | 266.4 | 14.95 |
| dhCer 8:0 | 85 | 25 | 428.2 | 266.2 | 8.27 |
| dhCer 6:0 | 65 | 20 | 400.1 | 266.1 | 5.33 |
| dhCer 28:1-1P | 80 | 45 | 786.3 | 266.2 | 21.61 |
| dhCer 28:0-1P | 80 | 55 | 788.3 | 266.2 | 22.93 |
| dhCer 26:1-1P | 70 | 45 | 758.2 | 266.2 | 24.29 |
| dhCer 26:0-1P | 75 | 40 | 760.2 | 266.2 | 16.91 |
| dhCer 24:1-1P | 60 | 35 | 730.2 | 266.2 | 21.17 |
| dhCer 24:1 | 55 | 35 | 650.3 | 266.2 | 23.82 |
| dhCer 24:0\|DHCer (d18:0)/C24:0 | 55 | 42.5 | 652.3 | 266.2 | 14.55 |
| dhCer 22:0-1P | 60 | 25 | 704.2 | 266.2 | 18.86 |
| dhCer 22:0 | 85 | 30 | 624.3 | 266.1 | 23.61 |
| dhCer 20:1-1P | 60 | 25 | 674.2 | 266.2 | 16.92 |
| dhCer 20:0 | 75 | 25 | 596.3 | 266.2 | 20.69 |
| dhCer 18:1 | 75 | 35 | 566.2 | 266.2 | 11.79 |
| dhCer 18:0-1P | 70 | 25 | 648.2 | 266.2 | 21.36 |
| dhCer 18:0(DHCer (d18:0)/C18:0) | 75 | 39 | 568.2 | 266.2 | 18.72 |
| dhCer 16:1-1P | 60 | 25 | 618.2 | 266.2 | 13.57 |
| dhCer 16:0-1P | 60 | 25 | 620.2 | 266.2 | 19.32 |
| dhCer 16:0 | 85 | 35 | 540.2 | 266.2 | 16.96 |
| dhCer (d18:1/18:1)-1P | 70 | 25 | 646.2 | 266.2 | 21.37 |
| DHCer (d18:0)/C24:1 | 55 | 42.5 | 650.9 | 266.4 | 23.81 |
| d18:1-So1P | 70 | 40 | 380.3 | 264.3 | 15.18 |
| d18:0-Sa1P | 45 | 20 | 382.3 | 284.3 | 12.99 |
| d18:0 DHS1P | 50 | 25 | 382.4 | 266.4 | 12.95 |
| d17:0-Sa1P | 40 | 15 | 368.3 | 270.3 | 9.06 |
| d17:0 DHS1P | 50 | 25 | 368.4 | 252.2 | 10.30 |
| Cer1P (d18:1)/C16:0 | 65 | 47.5 | 618.7 | 264.4 | 18.92 |
| Cer 8:0-1P | 90 | 25 | 506.1 | 264.2 | 21.02 |
| Cer 28:1-1P | 90 | 40 | 784.4 | 264.1 | 21.62 |
| Cer 28:0-1P | 80 | 50 | 786.4 | 264.1 | 21.61 |
| Cer 26:1-1P | 85 | 40 | 756.1 | 264.1 | 19.09 |
| Cer 26:0-1P | 90 | 45 | 758.4 | 264.1 | 23.57 |
| Cer 26:0 | 70 | 30 | 678.8 | 264.1 | 20.39 |
| Cer 24:1-1P | 75 | 35 | 728.2 | 264.1 | 17.01 |
| Cer 24:0-1P\|Cer1P (d18:1)/C24:0 | 75 | 35 | 730.3 | 264.1 | 15.49 |
| Cer 24:0\|Cer (d18:1)/C24:0 | 55 | 42 | 650.9 | 264.4 | 23.80 |
| Cer 22:1-1P | 70 | 35 | 700.2 | 264.1 | 15.47 |
| Cer 22:0-1P | 75 | 35 | 702.3 | 264.1 | 15.41 |
| Cer 20:1-1P | 70 | 35 | 672.2 | 264.1 | 14.17 |
| Cer 20:0-1P | 65 | 35 | 674.2 | 264.1 | 24.54 |
| Cer 20:0 | 60 | 38 | 594.4 | 264.1 | 20.60 |
| Cer 18:1-1P | 70 | 35 | 644.2 | 264.1 | 19.58 |
| Cer 18:1 | 65 | 35 | 564.4 | 264.1 | 16.68 |
| Cer 18:0-1P | 70 | 35 | 646.2 | 264.1 | 21.35 |
| Cer 16:1-1P | 65 | 25 | 616.1 | 264.1 | 14.60 |
| Cer 16:0-1P | 40 | 25 | 618.1 | 264.1 | 18.90 |
| Cer 14:1-1P | 60 | 25 | 588.2 | 264.1 | 14.73 |
| Cer 14:0-1P | 60 | 30 | 590.2 | 264.1 | 21.91 |
| Cer 14:0 | 70 | 33 | 510.4 | 264.1 | 14.91 |
| Cer 12:1-1P | 65 | 26 | 560.1 | 264.1 | 14.94 |
| Cer 12:0-1P\|Cer1P (d18:1)/C12:0 | 65 | 26 | 562.1 | 264.1 | 19.32 |
| Cer 10:1-1P | 70 | 25 | 532.1 | 264.1 | 5.07 |
| Cer 10:0-1P | 70 | 25 | 534.1 | 264.1 | 14.61 |
| Cer 10:0 | 70 | 15 | 454.2 | 264.1 | 5.75 |
| Cer (d18:1/26:1)\|C26:1-Cer | 70 | 35 | 676.5 | 264.1 | 25.02 |
| Cer (d18:1/24:1) | 80 | 40 | 648.3 | 264.3 | 23.79 |
| Cer (d18:1)/C25:0 | 55 | 45 | 664.9 | 264.4 | 22.57 |
| Cer (d18:1)/C18:0 | 60 | 35 | 566.3 | 264.3 | 18.25 |
| Cer (d18:1)/C12:0 | 70 | 30 | 482.4 | 264.1 | 13.71 |
| Cer (17:1) 24:1 | 80 | 10 | 634.5 | 250.1 | 22.04 |
| Cer (17:1) 18:0 | 65 | 30 | 552.4 | 250.1 | 14.71 |
| C26:1-GlcCer | 35 | 55 | 838.6 | 264.3 | 25.10 |
| C26:0-GlcCer | 35 | 55 | 840.6 | 264.3 | 16.60 |
| C24:1-GlcCer | 40 | 55 | 810.6 | 264.3 | 21.70 |
| C24:0-GlcCer | 35 | 45 | 812.6 | 264.3 | 24.94 |
| C24:0-dHCer | 50 | 32 | 652.7 | 634.7 | 25.10 |
| C24:0-Cer | 75 | 40 | 650.3 | 264.3 | 23.80 |
| C22:1-GlcCer | 40 | 47 | 782.6 | 264.3 | 21.00 |
| C22:1-Cer | 80 | 40 | 620.3 | 264.3 | 20.71 |
| C22:0-GlcCer | 40 | 50 | 784.6 | 264.3 | 21.60 |
| C22:0-Cer | 80 | 40 | 622.3 | 264.3 | 23.56 |
| C20:1-GlcCer | 40 | 47 | 754.6 | 264.3 | 18.60 |
| C20:1-Cer | 80 | 30 | 592.3 | 264.3 | 18.50 |
| C20:0-GlcCer | 40 | 47 | 756.6 | 264.3 | 19.07 |
| C2:0-Cer | 55 | 23 | 342.3 | 264.3 | 10.27 |
| C18:1-GlcCer | 40 | 47 | 726.6 | 264.3 | 16.73 |
| C18:0-GlcCer | 40 | 47 | 728.6 | 264.3 | 17.05 |
| C17:0-GlcCer | 40 | 45 | 714.5 | 264.5 | 15.62 |
| C16:1-Cer | 55 | 25 | 536.3 | 264.3 | 15.81 |
| C16:0-GlcCer | 35 | 45 | 700.6 | 264.3 | 15.44 |
| C16:0-dHCer | 50 | 25 | 540.5 | 522.5 | 16.96 |
| C16:0-Cer | 50 | 35 | 538.3 | 264.3 | 16.42 |
| C16:0-GlcCer | 35 | 45 | 714.5 | 262.3 | 14.14 |
| 3-KDS(d20:0) | 60 | 28 | 328.2 | 298.4 | 1.80 |
| 3-KDS(d19:0) | 60 | 26 | 314.2 | 284.4 | 8.60 |
| 3-KDS(d18:0) | 46 | 23 | 300 | 270.4 | 7.95 |
| 3-KDS(d17:0) | 60 | 22 | 286.2 | 256.4 | 8.70 |
| 3-KDS(d16:0) | 60 | 20 | 272.2 | 242.4 | 8.15 |
| d7-So d18:1 | 63 | 26 | 307.5 | 259.3 | 7.48 |
| d7-dhSph 18:0 | 65 | 30 | 309.5 | 261.3 | 7.9 |

## Table S4 Differential Metabolites Identified in salivary glands Tissue

| **Metabolite name** | **Fc (ESS/Cont)** | **P value** | **Molecular formula** |
| --- | --- | --- | --- |
| So(d20:1) | 0.8694 | 0.1376 | C_20_H_39_NO_2_ |
| So(d18:1) | 1.0836 | 0.6701 | C_18_H_37_NO_2_ |
| So(d16:1) | 0.8376 | 0.0008 | C_16_H_33_NO_2_ |
| SM(d18:1/24:1) | 0.9070 | 0.4953 | C_47_H_93_N_2_O_5_P |
| SM(d18:1/24:0) | 1.0712 | 0.6144 | C_47_H_95_N_2_O_5_P |
| SM(d18:1/22:0) | 0.8759 | 0.0688 | C_45_H_91_N_2_O_6_P |
| SM(d18:1/20:0) | 1.1165 | 0.2854 | C_43_H_88_N_2_O_6_P |
| SM(d18:1/18:1) | 0.8666 | 0.1388 | C_41_H_81_N_2_O_5_P |
| SM(d18:1/18:0) | 0.1518 | 0.0112 | C_41_H_83_N_2_O_5_P |
| SM(d18:1/16:0) | 0.9376 | 0.3280 | C_39_H_79_N_2_O_5_P |
| SM(d18:0/24:0) | 1.1406 | 0.4330 | C_47_H_97_N_2_O_5_P |
| SM(d18:0/16:0) | 0.9369 | 0.4239 | C_39_H_81_N_2_O_5_P |
| Sa(d20:0) | 0.8323 | 0.0215 | C_20_H_43_NO_2_ |
| Sa(d18:0) | 1.1692 | 0.0009 | C_18_H_39_NO_2_ |
| Sa(d17:0) | 0.8217 | 0.0001 | C_17_H_37_NO_2_ |
| Sa(d16:0) | 0.8255 | 0.0002 | C_16_H_35_NO_2_ |
| LacCer(d18:1/24:1) | 0.9012 | 0.5725 | C_52_H_97_NO_13_ |
| LacCer(d18:1/24:0) | 1.2099 | 0.2842 | C_52_H_100_NO_13_ |
| LacCer(d18:1/22:0) | 0.8808 | 0.5834 | C_50_H_97_NO_13_ |
| LacCer(d18:1/16:0) | 0.9814 | 0.9552 | C_44_H_85_NO_13_ |
| GluCer(d18:1/24:1) | 0.7299 | 0.2496 | C_48_H_91_NO_8_ |
| GluCer(d18:1/24:0) | 0.4076 | 0.3810 | C_48_H_93_NO_8_ |
| GluCer(d18:1/22:0) | 0.5890 | 0.4688 | C_46_H_89_NO_8_ |
| GluCer(d18:1/20:0) | 0.4231 | 0.4225 | C_44_H_85_NO_8_ |
| GluCer(d18:1/18:1) | 0.8978 | 0.7080 | C_42_H_81_NO_8_ |
| GluCer(d18:1/18:0) | 0.4086 | 0.4285 | C_42_H_83_NO_8_ |
| GluCer(d18:1/16:0) | 1.0093 | 0.9861 | C_40_H_79_NO_8_ |
| GluCer(d18:0/18:0) | 0.8324 | 0.1164 | C_42_H_83_NO_8_ |
| GluCer(d18:0/16:0) | 0.9895 | 0.9548 | C_40_H_81_NO_8_ |
| DHCer(d18:0/24:1(15Z)) | 0.7031 | 0.0806 | C_42_H_83_NO_3_ |
| DHCer(d18:0/24:0) | 0.7104 | 0.1881 | C_42_H_85_NO_3_ |
| DHCer(d18:0/22:0) | 0.8825 | 0.5806 | C_40_H_81_NO_3_ |
| DHCer(d18:0/20:0) | 0.8066 | 0.2892 | C_38_H_77_NO_3_ |
| DHCer(d18:0/18:1(9Z)) | 0.6752 | 0.0874 | C_36_H_73_NO_3_ |
| DHCer(d18:0/18:0) | 0.7688 | 0.1115 | C_36_H_73_NO_3_ |
| DHCer(d18:0/16:0) | 0.6406 | 0.0033 | C_34_H_69_NO_3_ |
| CerP(d18:1/22:0) | 0.2682 | 0.0038 | C_40_H_80_NO_5_P |
| CerP(d18:1/20:0) | 0.7792 | 0.4774 | C_38_H_76_NO_5_P |
| CerP(d18:1/18:1) | 0.8677 | 0.6777 | C_36_H_70_NO_5_P |
| CerP(d18:1/18:0) | 0.9603 | 0.8523 | C_36_H_72_NO_6_P |
| CerP(d18:1/16:0) | 0.8253 | 0.1231 | C_34_H_68_NO_5_P |
| CerP(d18:0/16:0) | 0.8220 | 0.3987 | C_34_H_72_NO_5_P |
| Cer(d18:1/6:0) | 0.8004 | 0.1623 | C_24_H_47_NO_3_ |
| Cer(d18:1/24:1(15Z)) | 0.6885 | 0.1621 | C_42_H_81_NO_3_ |
| Cer(d18:1/24:0) | 0.8131 | 0.2662 | C_42_H_83_NO_3_ |
| Cer(d18:1/22:0) | 0.7996 | 0.1734 | C_40_H_79_NO_3_ |
| Cer(d18:1/20:0) | 0.8146 | 0.2807 | C_38_H_75_NO_3_ |
| Cer(d18:1/18:0) | 0.6957 | 0.0801 | C_36_H_71_NO_3_ |
| Cer(d18:1/16:0) | 0.8428 | 0.5087 | C_34_H_67_NO_3_ |
| Cer(d18:1/14:0) | 0.7207 | 0.0442 | C_32_H_63_NO_3_ |

## Table S5 Differential Metabolites Identified in Lung Tissue

| **Metabolite name** | **Fc (ESS/Cont)** | **P value** | **Molecular formula** |
| --- | --- | --- | --- |
| So(d18:1) | 1.1493 | 0.3535 | C_18_H_37_NO_2_ |
| So(d16:1) | 1.3869 | 0.0004 | C_16_H_33_NO_2_ |
| SM(d18:1/22:0) | 0.8431 | 0.0366 | C_45_H_91_N_2_O_6_P |
| SM(d18:1/20:0) | 0.8663 | 0.0533 | C_43_H_88_N_2_O_6_P |
| SM (d18:1/24:1) | 0.8601 | 0.0914 | C_47_H_93_N_2_O_5_P |
| SM (d18:1/18:1) | 0.3048 | 0.0002 | C_41_H_81_N_2_O_5_P |
| SM (d18:1/18:0) | 0.8025 | 0.0236 | C_41_H_83_N_2_O_5_P |
| SM (d18:1/16:0) | 0.9134 | 0.1715 | C_39_H_79_N_2_O_5_P |
| SM (d18:1/12:0) | 0.7699 | 0.0609 | C_35_H_71_N_2_O_5_P |
| Sa(d20:0) | 1.1789 | 0.1732 | C_20_H_43_NO_2_ |
| Sa(d18:0) | 1.2496 | 0.0006 | C_18_H_39_NO_2_ |
| Sa(d16:0) | 1.2124 | 0.0008 | C_16_H_35_NO_2_ |
| S1P(d18:1) | 1.0711 | 0.6124 | C_18_H_38_NO_5_P |
| S1P(d18:0) | 1.0055 | 0.9613 | C_18_H_40_NO_5_P |
| S1P(d17:0) | 1.1049 | 0.5012 | C_17_H_38_NO_5_P |
| S1P(d16:0) | 0.8472 | 0.1862 | C_16_H_36_NO_5_P |
| PhytoSo | 1.0472 | 0.4218 | C_18_H_39_NO_3_ |
| LacCer(d18:1/24:1) | 0.9883 | 0.9161 | C_52_H_97_NO_13_ |
| LacCer(d18:1/22:0) | 0.9843 | 0.8985 | C_50_H_97_NO_13_ |
| LacCer(d18:1/20:0) | 1.0207 | 0.8643 | C_48_H_93_NO_13_ |
| LacCer(d18:1/18:0) | 1.0341 | 0.8244 | C_46_H_89_NO_13_ |
| LacCer(d18:1/16:0) | 1.2404 | 0.1154 | C_44_H_85_NO_13_ |
| GM3 24:1 | 0.6443 | 0.0288 | C_59_H_107_N_1_O_21_ |
| GM3 24:0 | 0.6759 | 0.0432 | C_59_H_109_N_1_O_21_ |
| GM3 22:0 | 0.6322 | 0.0250 | C_57_H_105_N_1_O_21_ |
| GM3 20:0 | 0.9502 | 0.6923 | C_55_H_101_N_1_O_21_ |
| GM3 16:0 | 0.6189 | 0.0137 | C_51_H_93_N_1_O_21_ |
| GluCer(d18:1/18:0) | 0.6145 | 0.3625 | C_42_H_83_NO_8_ |
| GluCer(d18:1/16:0) | 0.4714 | 0.2723 | C_40_H_79_NO_8_ |
| GluCer(d18:0/16:0) | 1.3078 | 0.0643 | C_40_H_81_NO_8_ |
| GlcCer(d18:1/24:1) | 0.7719 | 0.4475 | C_48_H_93_NO_8_ |
| GlcCer(d18:1/24:0) | 0.6496 | 0.3429 | C_48_H_95_NO_8_ |
| GlcCer(d18:1/22:1) | 0.3315 | 0.2947 | C_46_H_89_NO_8_ |
| GlcCer(d18:1/22:0) | 0.6555 | 0.3524 | C_46_H_91_NO_8_ |
| GlcCer(d18:1/20:1) | 0.3787 | 0.3043 | C_44_H_85_NO_8_ |
| GlcCer(d18:1/20:0) | 0.5351 | 0.3553 | C_44_H_87_NO_8_ |
| DHGlcCer (d18:0)/C18:0 | 0.9310 | 0.5517 | C_42_H_85_NO_8_ |
| DHGlcCer (d18:0)/C16:0 | 0.8812 | 0.1952 | C_40_H_81_NO_8_ |
| DHGlcCer (d18:0)/C12:0 | 1.1816 | 0.1822 | C_36_H_73_NO_8_ |
| DHCer1P (d18:1/18:0) | 1.2841 | 0.0555 | C_36_H_72_NO_5_P |
| DHCer1P (d18:0/24:1) | 0.8858 | 0.4698 | C_42_H_84_NO_5_P |
| DHCer1P (d18:0/24:0) | 0.9280 | 0.6310 | C_42_H_86_NO_5_P |
| DHCer1P (d18:0/22:0) | 0.9795 | 0.8426 | C_40_H_82_NO_5_P |
| DHCer1P (d18:0/20:1) | 1.0059 | 0.9645 | C_38_H_78_NO_5_P |
| DHCer1P (d18:0/16:0) | 0.9178 | 0.3690 | C_34_H_70_NO_5_P |
| DHCer(d18:0/8:0) | 0.8328 | 0.3131 | C_26_H_53_NO_3_ |
| DHCer(d18:0/24:1) | 1.0609 | 0.6065 | C_42_H_83_NO_3_ |
| DHCer(d18:0/24:1(15Z)) | 1.0571 | 0.6103 | C_42_H_83_NO_3_ |
| DHCer(d18:0/24:0) | 0.9628 | 0.8496 | C_42_H_85_NO_3_ |
| DHCer(d18:0/22:0) | 1.0634 | 0.5138 | C_40_H_81_NO_3_ |
| DHCer(d18:0/20:0) | 0.9173 | 0.4978 | C_38_H_77_NO_3_ |
| DHCer(d18:0/18:0) | 1.0853 | 0.4060 | C_36_H_73_NO_3_ |
| DHCer(d18:0/16:1) | 1.1863 | 0.1238 | C_34_H_67_NO_3_ |
| DHCer(d18:0/16:0) | 1.1545 | 0.2116 | C_34_H_69_NO_3_ |
| CerP(d18:1/8:0) | 0.9792 | 0.9116 | C_26_H_52_NO_5_P |
| CerP(d18:1/28:1) | 0.6475 | 0.3175 | C_46_H_90_NO_5_P |
| CerP(d18:1/24:1) | 0.6331 | 0.3209 | C_42_H_82_NO_5_P |
| CerP(d18:1/22:1) | 1.2742 | 0.1195 | C_40_H_78_NO_5_P |
| CerP(d18:1/22:0) | 1.2646 | 0.1359 | C_40_H_80_NO_5_P |
| CerP(d18:1/20:0) | 1.2736 | 0.1421 | C_38_H_76_NO_5_P |
| CerP(d18:1/18:1) | 1.6473 | 0.0082 | C_36_H_70_NO_5_P |
| CerP(d18:1/18:0) | 1.2168 | 0.1470 | C_36_H_72_NO_6_P |
| CerP(d18:1/16:0) | 1.1143 | 0.4282 | C_34_H_68_NO_5_P |
| CerP(d18:1/14:0) | 1.0833 | 0.4307 | C_32_H_64_NO_5_P |
| CerP(d18:1/12:0) | 0.9914 | 0.9432 | C_30_H_60_NO_5_P |
| CerP(d18:1/10:0) | 1.0491 | 0.7536 | C_28_H_56_NO_5_P |
| Cer(d18:1/24:1(15Z)) | 1.0570 | 0.6046 | C_42_H_81_NO_3_ |
| Cer(d18:1/24:0) | 0.8591 | 0.1840 | C_42_H_83_NO_3_ |
| Cer(d18:1/22:1) | 1.0650 | 0.6089 | C_40_H_77_NO_3_ |
| Cer(d18:1/22:0) | 1.0387 | 0.7479 | C_40_H_79_NO_3_ |
| Cer(d18:1/20:1) | 0.8308 | 0.5840 | C_38_H_73_NO_3_ |
| Cer(d18:1/20:0) | 1.0469 | 0.6817 | C_38_H_75_NO_3_ |
| Cer(d18:1/18:1) | 1.0480 | 0.7870 | C_36_H_69_NO_3_ |
| Cer(d18:1/18:0) | 0.9646 | 0.7032 | C_36_H_71_NO_3_ |
| Cer(d18:1/16:0) | 1.1316 | 0.1984 | C_34_H_67_NO_3_ |
| Cer(d18:1/14:0) | 0.9784 | 0.8636 | C_32_H_63_NO_3_ |
| Cer(d18:1/10:0) | 0.9621 | 0.6892 | C_28_H_55_NO_3_ |
| Cer(d17:1/18:0) | 1.3374 | 0.0594 | C_35_H_69_NO_3_ |
| 3-KDS(d20:0) | 1.7115 | 0.0351 | C_20_H_41_NO_2_ |

# Supplementary figures

## Figure S1
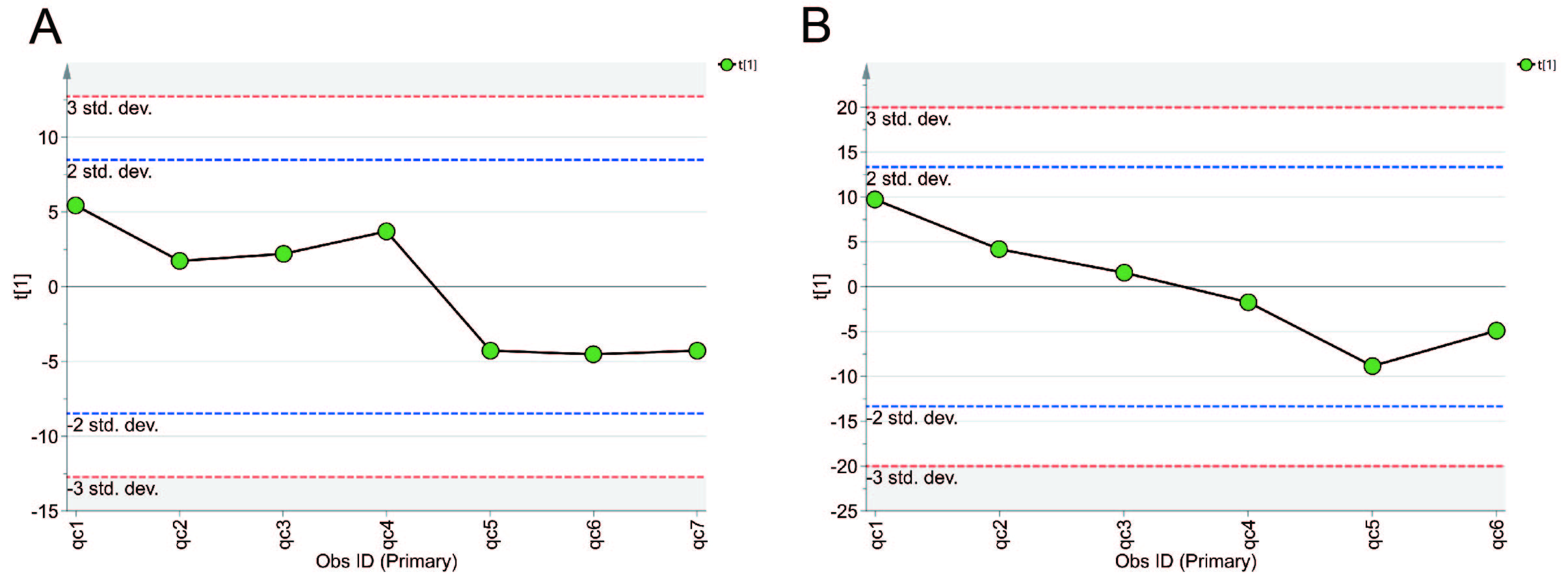


Figure S1 The PCA line score plots of quality control (QC) samples in positive **(A)**, negative ion mode **(B)**.

## Figure S2


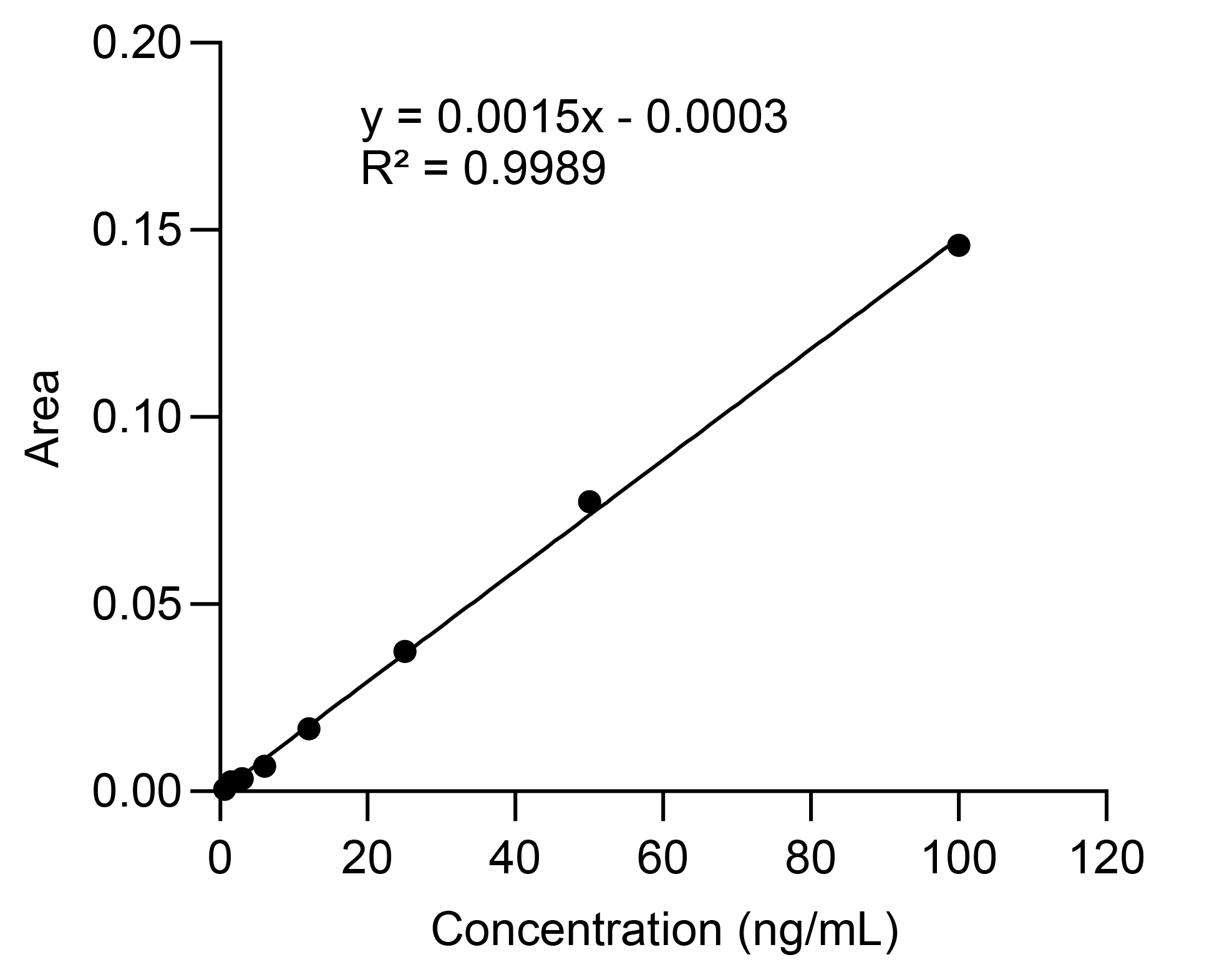


**Figure S2. Calibration curve of sphinganine.** Standard curve for sphinganine (0–100 ng/mL), with linear regression equation *y = 0.0015x − 0.0003* and *R² = 0.9989*. This curve was used for absolute quantification.

## **Figure S3**


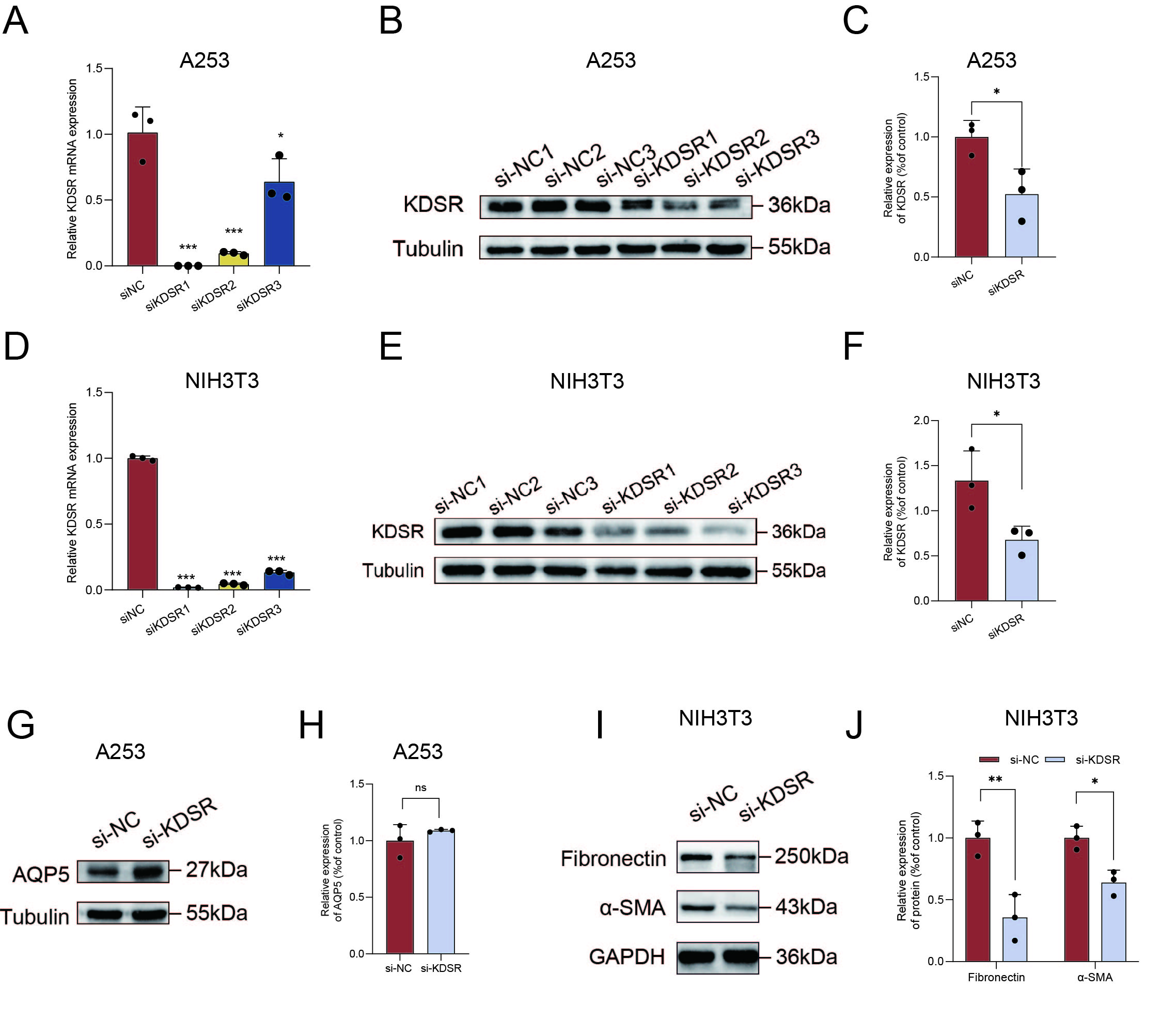


**Figure S3 Knockdown of KDSR reduces sphinganine synthesis and attenuates epithelial injury and fibroblast activation.**
(A–C) Validation of KDSR knockdown in A253 cells by qRT-PCR (A), Western blot (B), and densitometric analysis (C). (D–F) KDSR silencing efficiency in NIH3T3 cells confirmed by qRT-PCR (D), Western blot (E), and quantification (F). (G–H) Western blot (G) and quantification (H) showing that KDSR knockdown does not significantly affect AQP5 expression in A253 cells. In NIH3T3 fibroblasts, KDSR knockdown reduces fibronectin and α-SMA expression, as shown by Western blot (I) and quantitative analysis (J).

Data are presented as mean ± SD. *P < 0.05, **P < 0.01, ***P < 0.001 vs. si-NC; ns, not significant.

## **Figure S4**


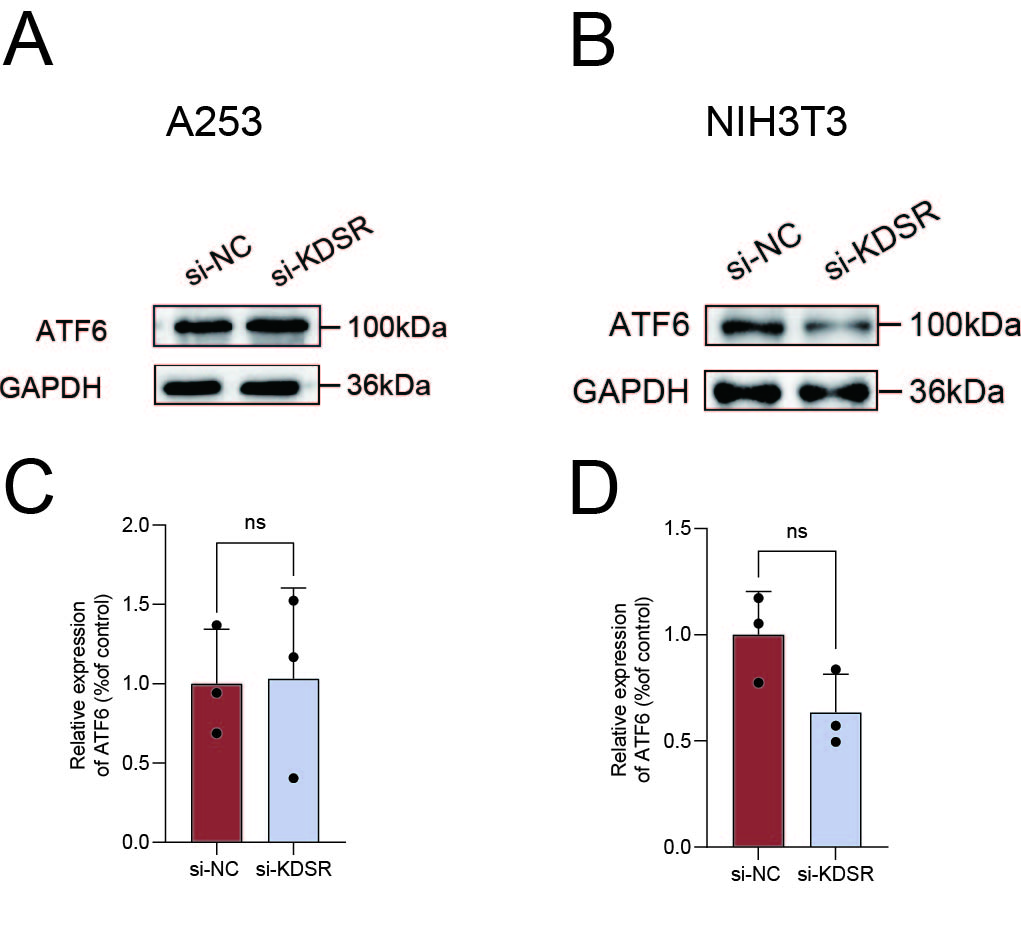


**Figure S4. Effect of KDSR knockdown on ATF6 expression in A253 and NIH3T3 cells.**

Western blot analysis of ATF6 protein expression in A253 cells (A) and NIH3T3 fibroblasts (B) treated with si-NC or si-KDSR. Quantification of ATF6 protein levels normalized to GAPDH in A253 (C) and NIH3T3 cells (D), indicating no significant change following KDSR knockdown. *Data are presented as mean ± SD. ns, not significant vs. si-NC*

## **Figure S5**


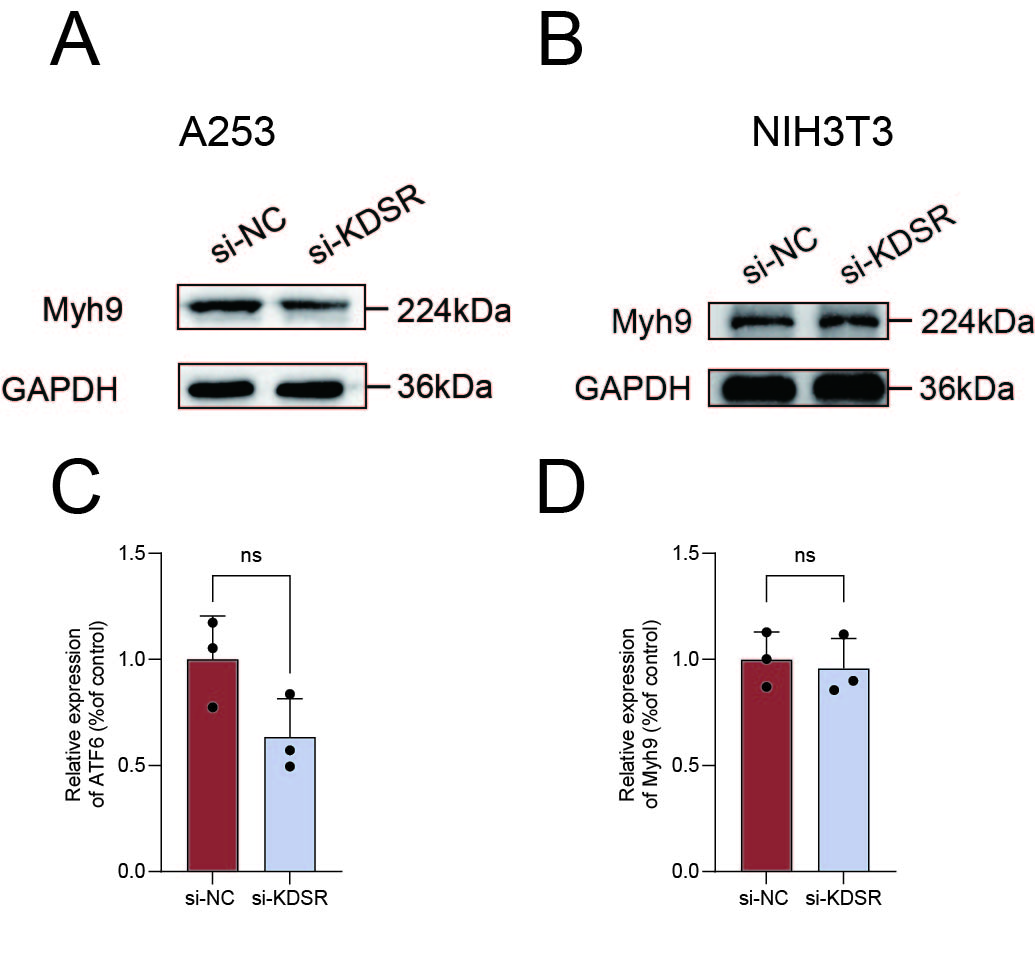


**Figure S5. Effect of KDSR knockdown on Myh9 expression in A253 and NIH3T3 cells.**

Western blot analysis of Myh9 protein expression in A253 cells (A) and NIH3T3 fibroblasts (B) treated with si-NC or si-KDSR. Quantification of Myh9 protein levels normalized to GAPDH in A253 (C) and NIH3T3 cells (D), indicating no significant change following KDSR knockdown. *Data are presented as mean ± SD. ns, not significant vs. si-NC*

## **Figure S6**


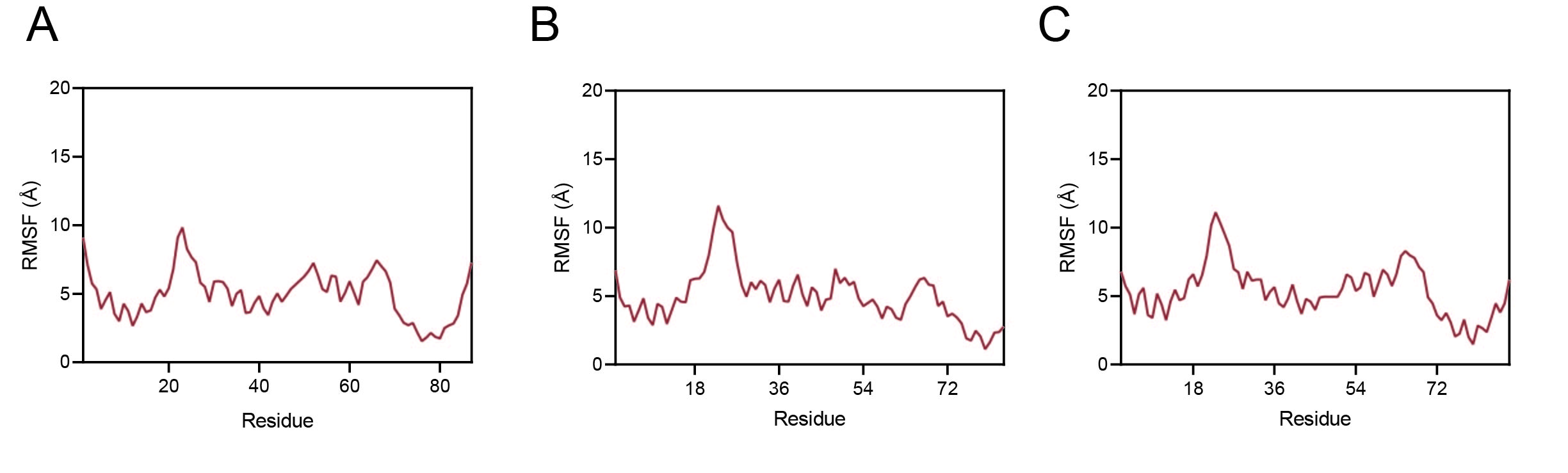


**Figure S6** RMSF profiles of chains A–C showing residue-level flexibility during simulation.

## **Figure S7**


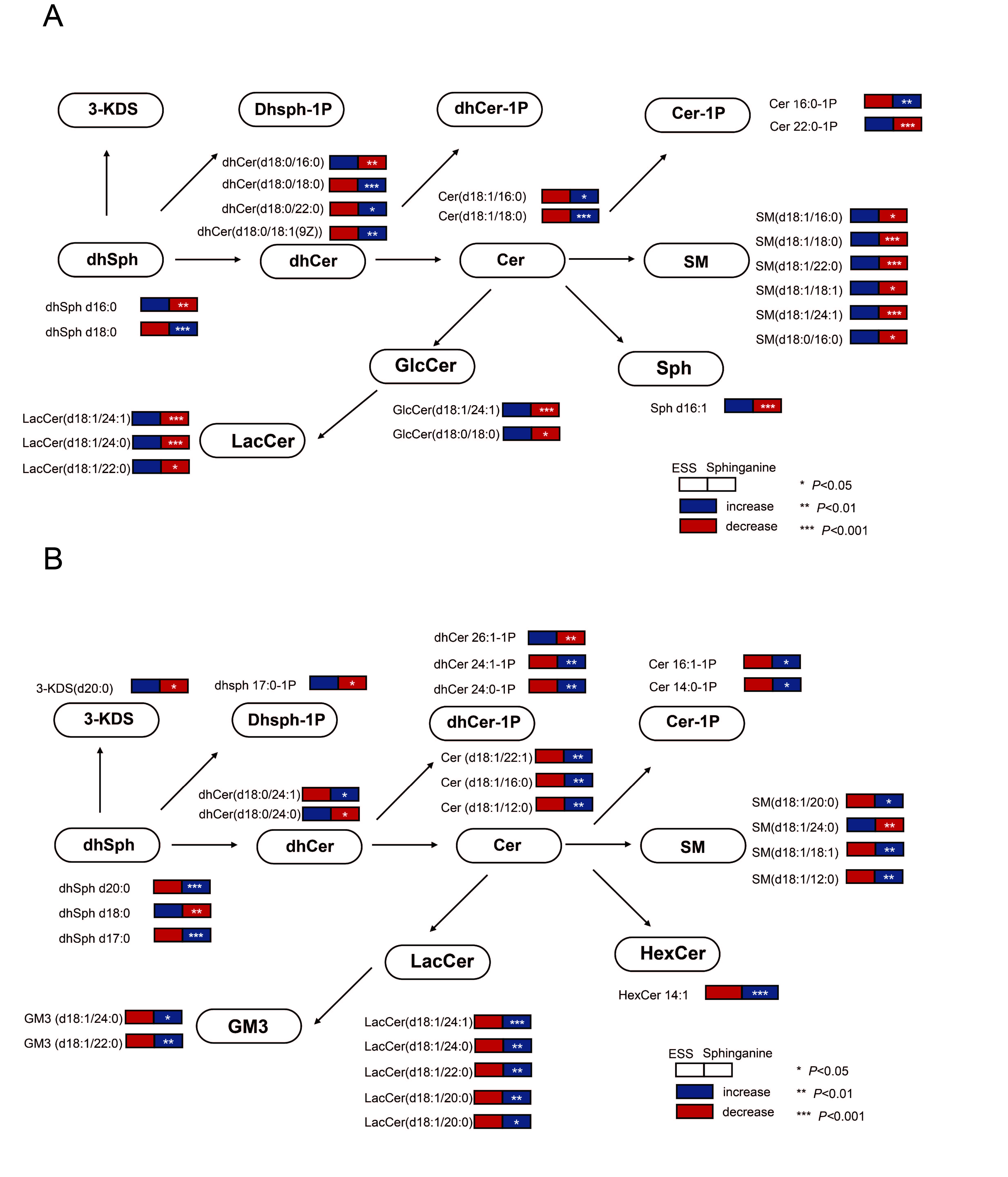


**Figure S7** **Sphinganine-induced alterations in sphingolipid metabolic pathways in submandibular gland and lung tissues.**
**(A)** Differential sphingolipid metabolites in the submandibular gland following sphinganine treatment compared with the ESS group. Red bars indicate increased levels, while blue bars indicate decreased levels in the sphinganine-treated group relative to ESS. **(B)** Differential sphingolipid metabolites in the lung under the same comparison. Statistical significance is marked as **P* < 0.05, ***P* < 0.01, ****P* < 0.001. Abbreviations: 3-KDS, 3-Dehydrosphinganine; dhSph, sphinganine; dhCer, dihydroceramide; Cer, ceramide; SM, sphingomyelin; GlcCer, glucosylceramide; LacCer, lactosylceramide, dhsph-1P, Sphinganine 1-phosphate; dhcer-1P, dihydroceramide 1-phosphate; cer-1P, ceramide 1-phosphate.
