# Supplementary figures and images for: Sphinganine as a potentially relevant metabolite in pulmonary involvement of primary Sjögren’s syndrome
Source: J Lipid Res. 2025 Dec 11;67(1):100961. doi: 10.1016/j.jlr.2025.100961 (PMC12814850; doi:10.1016/j.jlr.2025.100961)

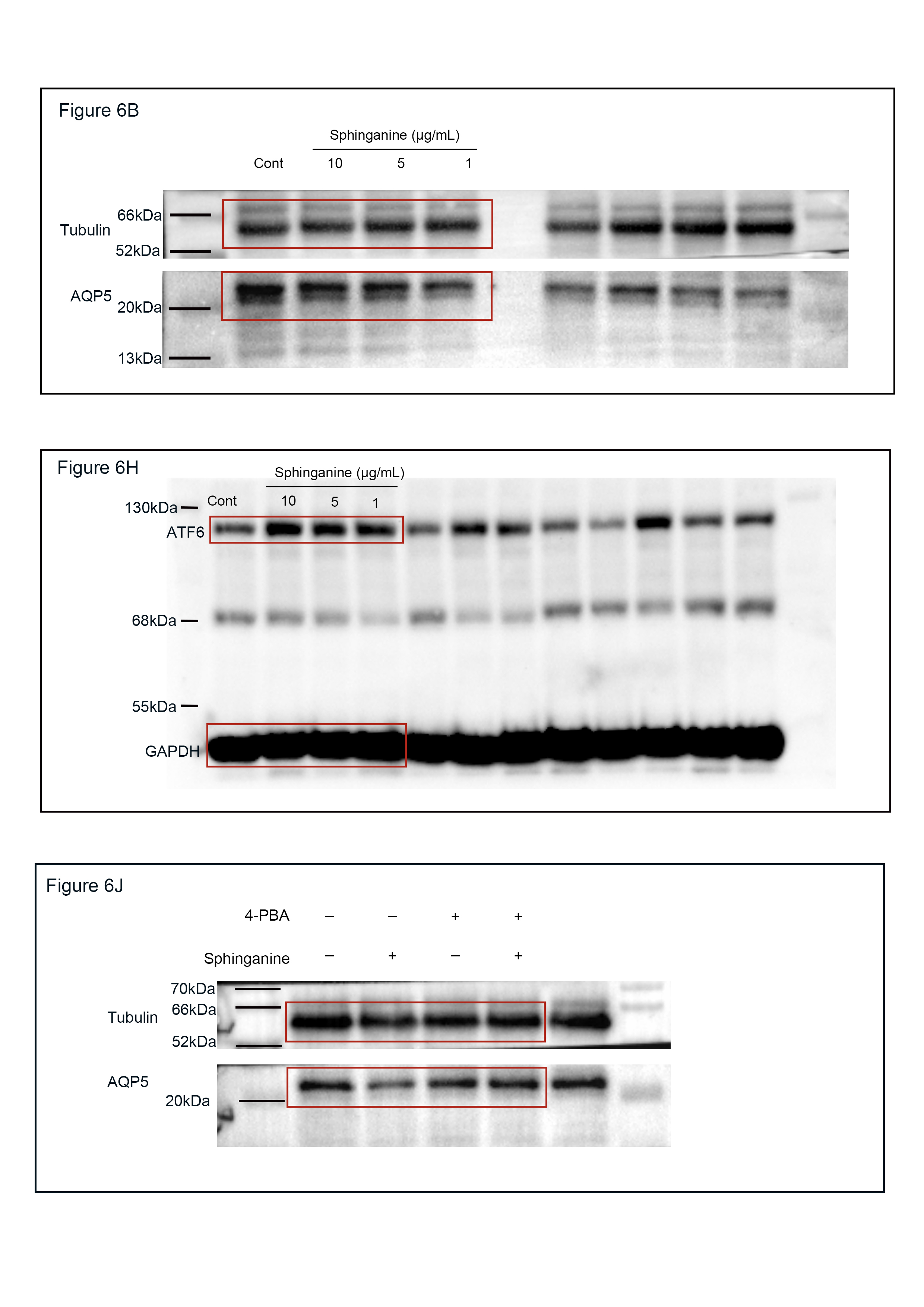

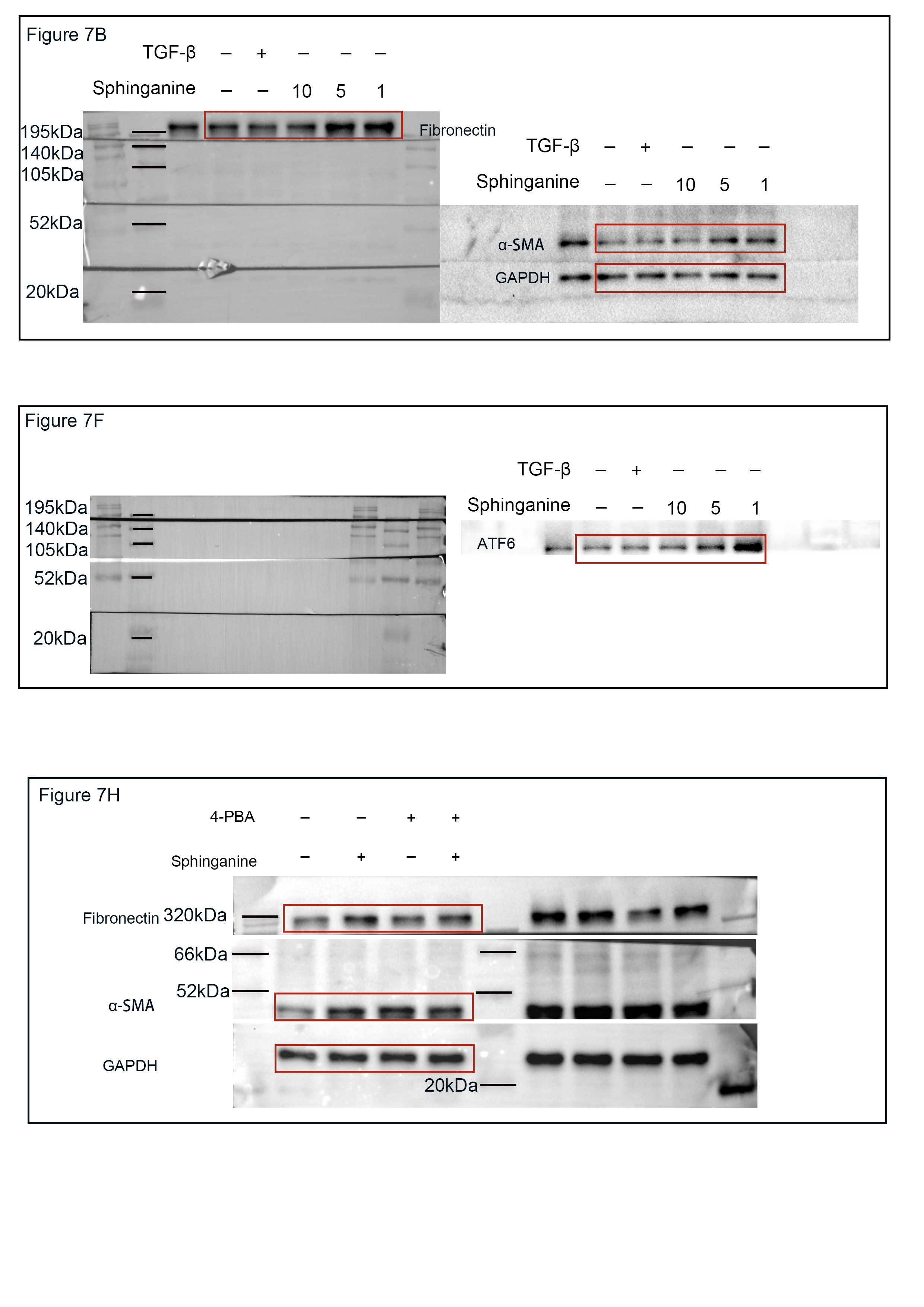

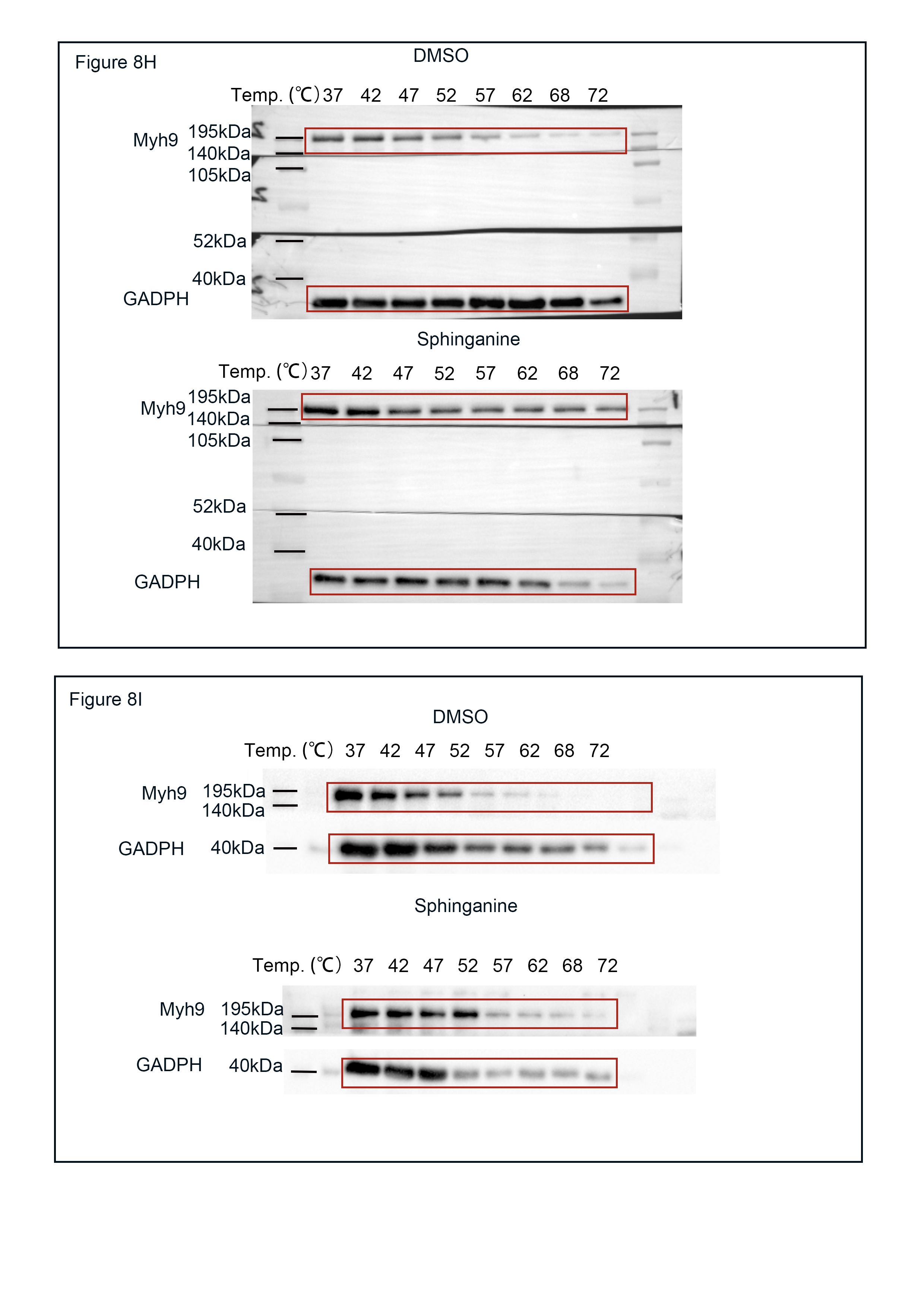

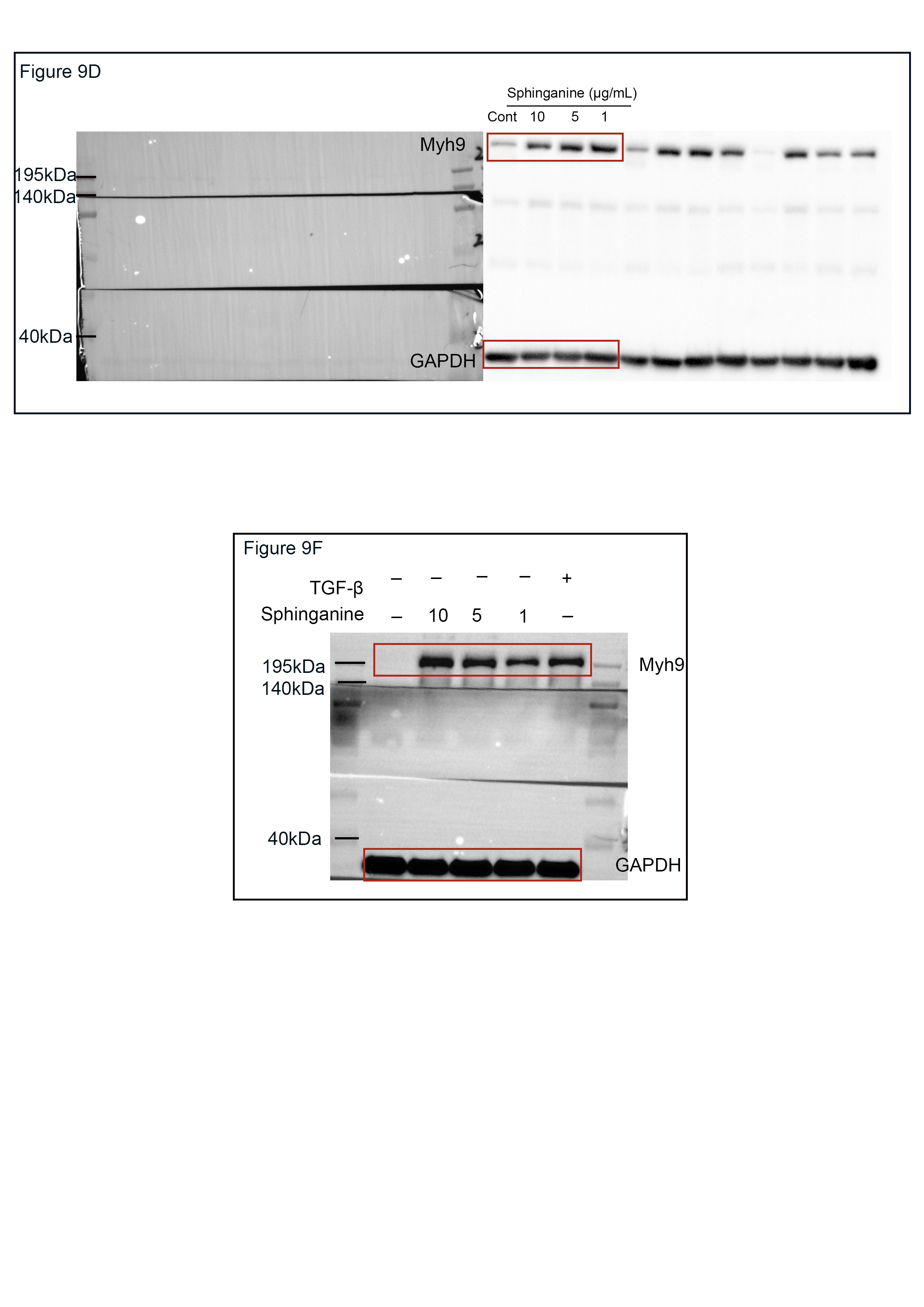

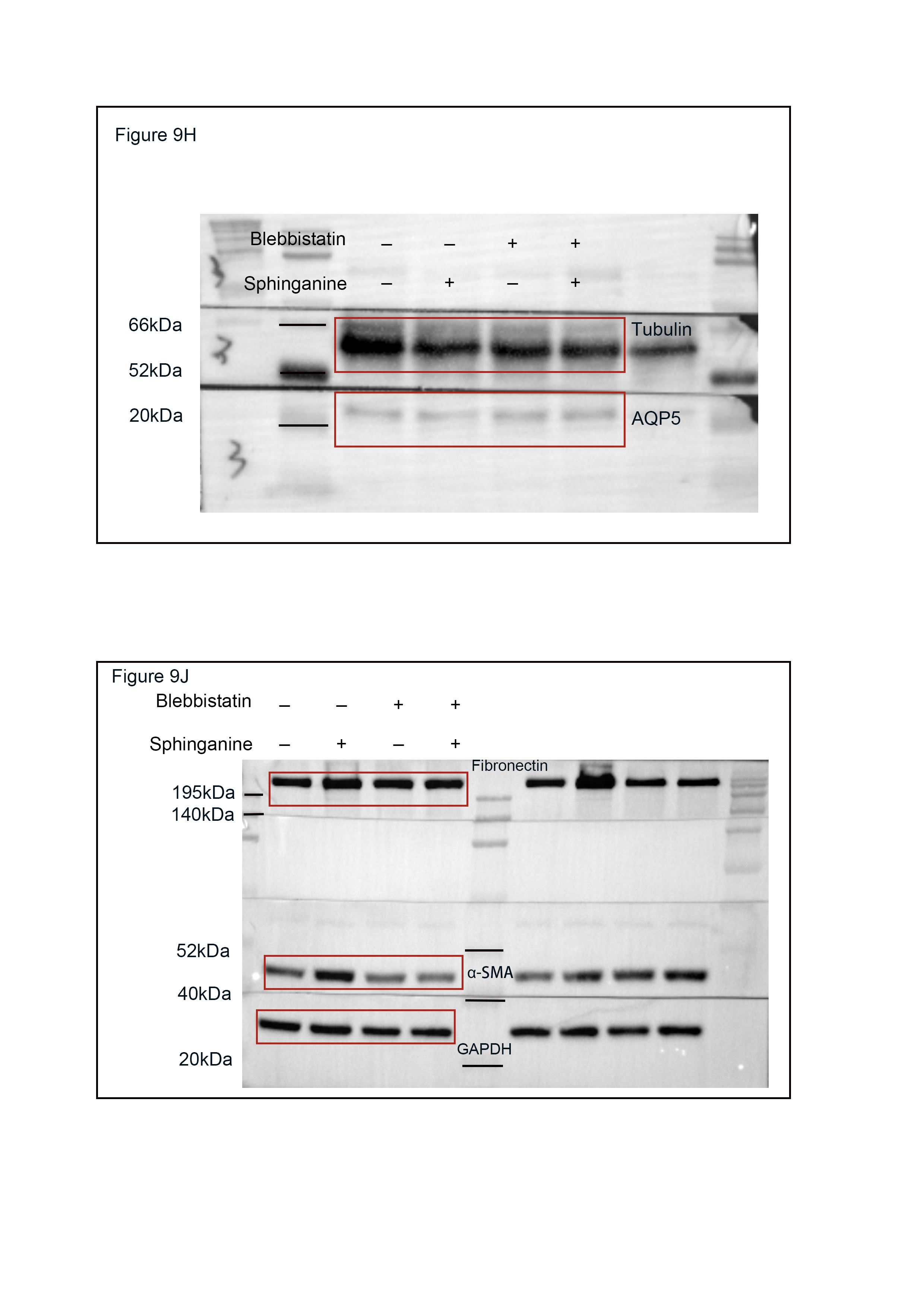

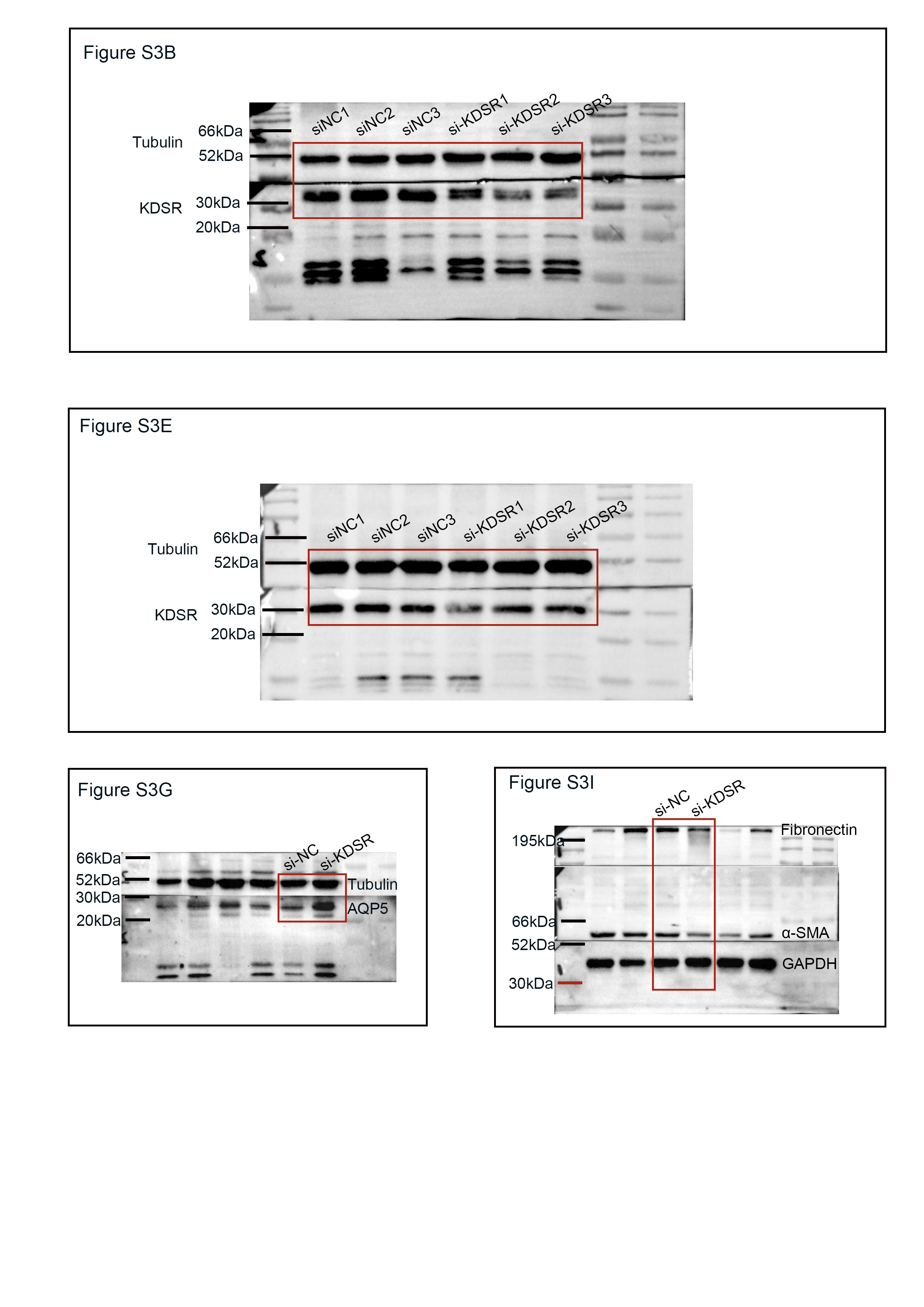

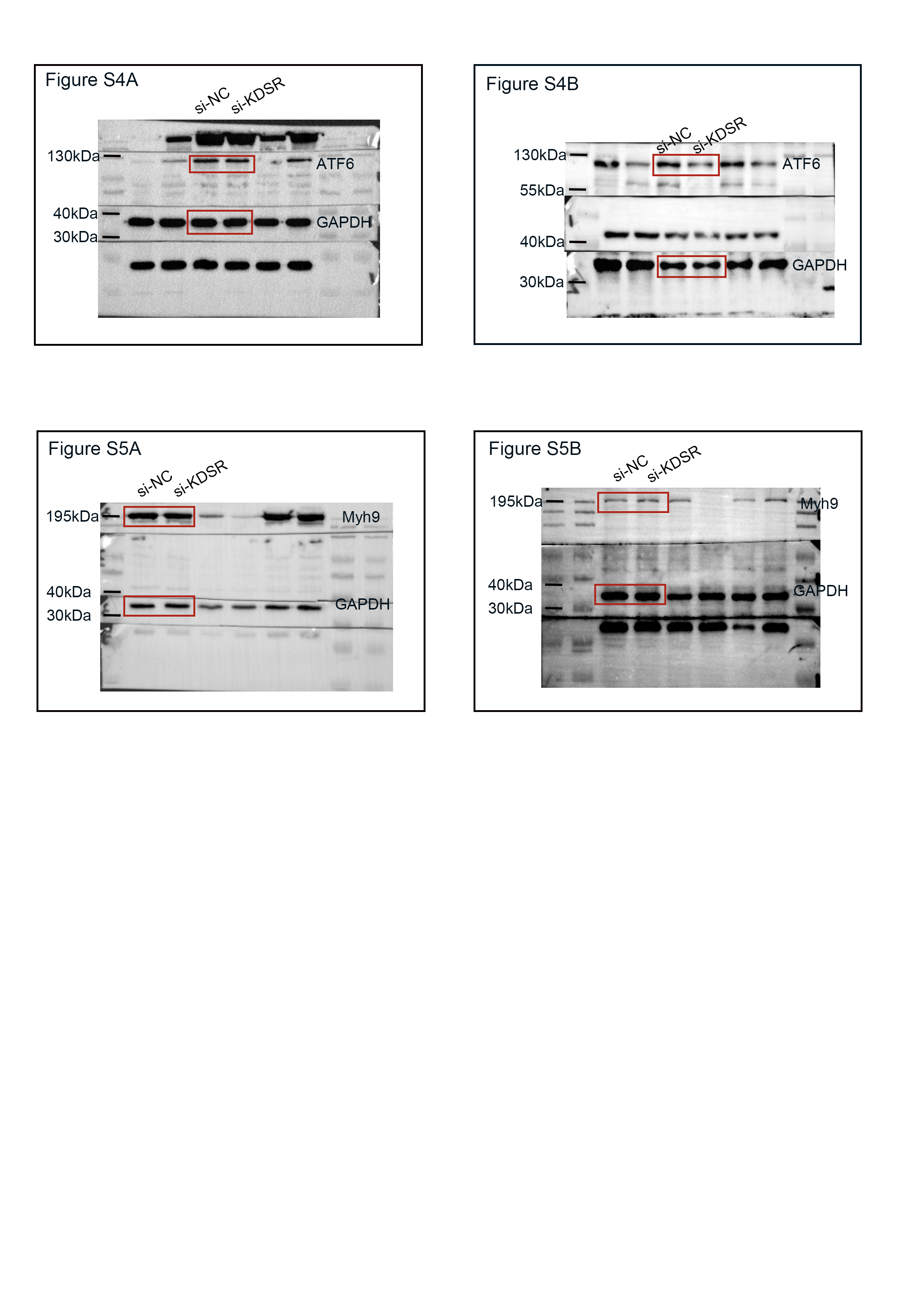

Supplement: Supplementary Material 2 [file mmc2.docx]
